# An Abrupt Decline in Global Terrestrial Water Storage and Its Relationship with Sea Level Change

**DOI:** 10.1007/s10712-024-09860-w

**Published:** 2024-11-04

**Authors:** Matthew Rodell, Anne Barnoud, Franklin R. Robertson, Richard P. Allan, Ashley Bellas-Manley, Michael G. Bosilovich, Don Chambers, Felix Landerer, Bryant Loomis, R. Steven Nerem, Mary Michael O’Neill, David Wiese, Sonia I. Seneviratne

**Affiliations:** 1 NASA Goddard Space Flight Center, Greenbelt, MD 20771 USA; 2 Magellium, 31520 Ramonville Saint-Agne, France; 3 NASA Marshall Space Flight Center, Huntsville, AL 35808 USA; 4 Department of Meteorology and National Centre for Earth Observation, University of Reading, Reading, RG6 6UR UK; 5 University of Colorado, Boulder, CO 80309 USA; 6 University of South Florida, Tampa, FL 33620 USA; 7 Jet Propulsion Laboratory, California Institute of Technology, Pasadena, CA 91011 USA; 8 University of Maryland, College Park, MD 20742 USA; 9 ETH Zurich, 8092 Zurich, Switzerland

**Keywords:** Climate change, Terrestrial water storage, Sea level, GRACE

## Abstract

As observed by the Gravity Recovery and Climate Experiment (GRACE) and GRACE Follow On (GRACE-FO) missions, global terrestrial water storage (TWS), excluding ice sheets and glaciers, declined rapidly between May 2014 and March 2016. By 2023, it had not yet recovered, with the upper end of its range remaining 1 cm equivalent height of water below the upper end of the earlier range. Beginning with a record-setting drought in northeastern South America, a series of droughts on five continents helped to prevent global TWS from rebounding. While back-to-back El Niño events are largely responsible for the South American drought and others in the 2014–2016 timeframe, the possibility exists that global warming has contributed to a net drying of the land since then, through enhanced evapotranspiration and increasing frequency and intensity of drought. Corollary to the decline in global TWS since 2015 has been a rise in barystatic sea level (i.e., global mean ocean mass). However, we find no evidence that it is anything other than a coincidence that, also in 2015, two estimates of barystatic sea level change, one from GRACE/FO and the other from a combination of satellite altimetry and Argo float ocean temperature measurements, began to diverge. Herein, we discuss both the mechanisms that account for the abrupt decline in terrestrial water storage and the possible explanations for the divergence of the barystatic sea level change estimates.

**Supplementary Information:**

The online version contains supplementary material available at 10.1007/s10712-024-09860-w.

**Article Highlights**

- Global terrestrial water storage, excluding glaciers and ice sheets, declined abruptly between May 2014 and March 2016, with a corollary increase in sea level
- A series of droughts, possibly linked to global warming, has since helped to prevent global terrestrial water storage from recovering
- Also around 2015, two independent estimates of barystatic sea level began to diverge, but we find no evidence of a connection with the terrestrial water storage decline

## Introduction

Terrestrial water storage (TWS; i.e., the sum of groundwater, soil moisture, surface waters, snow water equivalent, and ice) is an Essential Climate Variable (https://gcos.wmo.int/en/essential-climate-variables/tws) and a natural resource vital to ecosystems and societies. It exhibits substantial variability seasonally and over longer periods due to climate change and human water usage (Rodell et al. 2018; Intergovernmental Panel On Climate Change 2023). Some TWS variations are better understood in terms of physical processes than others, and understanding is limited by a satellite observational record (2002–present) that is short relative to most in situ data records and model analyses. In this study, we investigate an apparent abrupt decline in global, unfrozen TWS during 2014–2016 and a simultaneous divergence of independent estimates of changes in barystatic sea level (BSL, sometimes called global mean ocean mass), based on a combination of satellite data and observations-forced model and reanalysis output.

Observed changes in water stored on and in the land surface are balanced almost perfectly by changes in water stored in the ocean and atmosphere. There is a net loss of water from the ocean to the mantle, which is on the order of 0.4 to 1.3 GT/yr (Bounama et al. 2001). To put that into context, eight estimates of the linear trend in BSL based on Gravity Recovery and Climate Experiment (GRACE) (Tapley et al. 2004) and GRACE Follow On (GRACE-FO) (Landerer et al. 2020) satellite gravimetry measurements, an ensemble compiled by Chen et al. (2020), had a standard deviation of 0.20 mm/yr. Using the conversion rate of 360 GT water (~ 360 km^3^ water) per 1.0 mm sea level change, that equates to 72 GT/yr uncertainty in BSL change. The net loss of water to the mantle is at least an order of magnitude smaller. There is a tiny net loss of water (as hydrogen) from the stratosphere to space, less than 1 GT per 1000 years (Bounama et al. 2001). Ignoring rare impacts of > 1000 kg meteors, Earth’s current accretion rate of extraterrestrial material is smaller still, 0.049–0.056 GT per 1000 years (Esser and Turekian 1988; Love and Brownlee 1993), of which only a fraction may be water. Ergo, on timescales of seconds to centuries, it is appropriate to assume that the global water cycle is a closed system in which the law of conservation of mass applies. Multi-annual changes in global atmospheric water storage are small compared with those in ocean or land water mass, though not necessarily negligible. Trent et al. (2023) compared multiple sources of atmospheric water storage variations and trends, finding that recent trends are less than 0.5 mm/decade (excluding one outlier estimate). Regional variations can be larger owing to significant modes of variability (e.g., ENSO) and human-induced climate change (Intergovernmental Panel On Climate Change 2023), with local variations of ± 3 mm. Changes in atmospheric water vapor are substantially constrained by the Clausius-Clapeyron relationship (Trenberth et al. 2005; Allan et al. 2022; Intergovernmental Panel On Climate Change 2023) to small long-term trends at the global scale. Though small, global atmospheric moisture storage changes may be non-negligible when balancing TWS against ocean mass changes over decadal timescales and a source of error if ignored. Polar ice sheet and major glacier system mass changes are often separated from the remaining TWS in sea level budget accounting. Hereafter, unless otherwise noted, we define TWS as the aggregate of groundwater, soil moisture, surface waters, and ephemeral snow and ice (i.e., excluding ice sheets and major glacier systems).

Changes in global mean sea level (GMSL) comprise both the barystatic component due to ocean mass changes and the thermosteric component related to the temperature and salinity of the ocean water (Gregory et al. 2019). The rate of sea level rise during 1993–2017 was about 3.0 mm/yr—roughly 55% thermosteric and 45% barystatic gains (Nerem et al. 2018). Most of the barystatic gain is attributed to ablation of polar ice sheets and glaciers. GRACE and GRACE-FO (collectively, GRACE/FO) data indicate that the Greenland and Antarctic ice sheets have contributed 261 ± 45 GT/yr and 104 ± 57 GT/yr to BSL since 2002 (Velicogna et al. 2020). The rest of the world’s glacier systems contribute another 199 ± 32 GT/yr to BSL, including 53 ± 14 GT/yr from the glaciers along the gulf coast of Alaska (Wouters et al. 2019). While ice loss is the dominant driver of ocean mass change, Cáceres et al. (2020) showed that non-ice TWS is the primary control on seasonal and interannual variations of the land–ocean water balance excluding Greenland and Antarctica.

Shortly after launching in 2002, GRACE’s measurements of time variable gravity changes proved valuable for elucidating characteristics of the mass balance between ocean and land, including changes in TWS (Chambers et al. 2004; Chen et al. 2005; Ramillien et al. 2008; Cazenave and Llovel 2010; Riva et al. 2010). More recently, Wada et al. (2016) focused on the contribution of groundwater pumping and depletion to sea level rise. Chandanpurkar et al. (2021) investigated the amplitudes of seasonal exchanges of water between the global land and oceans during the GRACE/FO era, which average about 17 mm equivalent sea level with significant interannual variability. GRACE allowed scientists to explain that the unusual decline in global mean sea level during 2010–2011 was caused by a massive increase in TWS, largely in Australia (Boening et al. 2012; Fasullo et al. 2013). Rodell et al. (2015) and L’Ecuyer et al. (2015) constrained estimates of mean monthly water and energy cycle fluxes at continental and global scales by enforcing water and energy budget closure, with GRACE-based estimates of seasonal changes in land and ocean water storage playing a key role. Of particular relevance, Reager et al. (2016) calculated that regional increases in precipitation during 2002–2014 had raised global TWS to the extent that the rate of sea level rise was reduced by about 15% after removing the effects of irrigation-enhanced groundwater depletion. Rietbroek et al. (2016) obtained similar results. However, improvements in two key sources of auxiliary information used in GRACE/FO data processing (a glacial isostatic adjustment (GIA) model and the determination of the C_20_ spherical harmonic coefficient based on satellite laser ranging data) flipped what had been a 0.32 mm/yr sea level equivalent increase in TWS to a 0.09 mm/yr decrease over the period of April 2002 to November 2014, noting that this magnitude of change is within a standard two-sigma uncertainty range for systematic errors (Chambers et al. 2017).

**Fig. 1:**
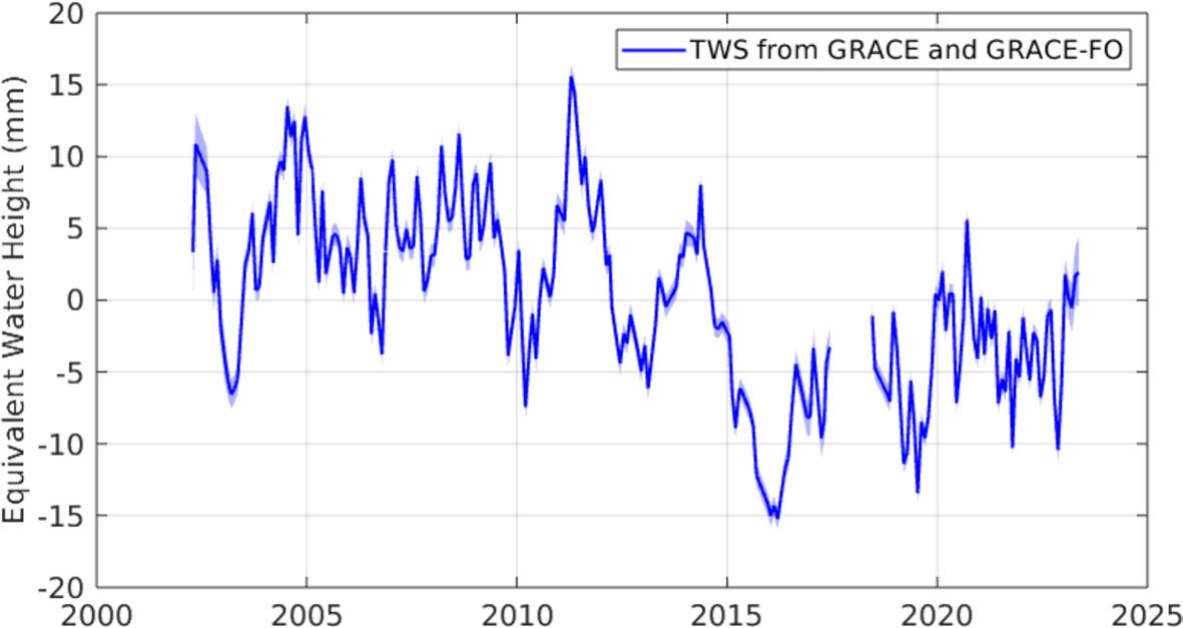
Anomalies (relative to the 2003–2020 mean) of global mean terrestrial water storage (equivalent height of water over land) from GRACE and GRACE-FO after removing annual and semiannual components and the S2 and K2 tidal alias terms. Ice covered land regions excluded from the global mean are shown in Figure S1. The shading conveys the formal error estimates, which average about ± 0.9 mm

The timeframe of the latter two studies, 2002–2014, immediately preceded a rapid decrease and two decade minimum in TWS that is the focus of the present study. As shown in Fig. 1, global mean TWS anomalies (deviations from the 2003–2020 average) remained within the range -8 to + 16 mm from the onset of observations until the end of 2014. By the end of 2015, a new record minimum of – 15 mm had been set, and the upper end of the range in the following years was about + 6 mm. The April 2002 to December 2014 average anomaly was + 33 mm, and the January 2015 to May 2023 average was – 51 mm. As detailed in the Results, the two independently derived estimates of BSL, one from GRACE/FO and the other from satellite altimetry minus Argo float based thermosteric sea level change, began to diverge, also around 2015, after being generally consistent beforehand (Barnoud et al. 2021). This led to speculation in the climate change community about errors or drift in one or more of the observational time series. The main purpose of the present study is to investigate the abrupt decline in TWS. We also discuss uncertainties in the various contributing datasets that may have led to the divergence between the direct (from GRACE/FO) and indirect (altimeter minus Argo) BSL estimates of global mean thermosteric sea level change, but we do not offer a definitive conclusion.

## Data and Methods

### GRACE and GRACE-FO

GRACE (2002–2017) was a twin satellite mission that produced global, monthly fields of surface gravitational and (equivalently) mass anomalies. Micron-scale microwave-ranging measurements of changes in the distance between the two satellites (nominally around 200 km) as they orbited the Earth were combined with precise location and onboard accelerometer data to infer surface mass effects on the orbits and hence to derive the global anomaly fields (Wahr et al. 1998; Tapley et al. 2004). GRACE-FO (2018-present) is nearly identical to GRACE, with the addition of a laser ranging instrument (Landerer et al. 2020). The mass anomaly data provided by both missions have proven valuable for climatology, oceanography, cryoscience, carbon cycle science, and hydrology (Humphrey et al. 2018, 2023; Tapley et al. 2019; Flechtner et al. 2021; Rodell and Reager 2023).

We used the Jet Propulsion Laboratory RL06.1Mv03 GRACE/GRACE-FO mascon solution for our primary estimates of TWS anomalies (Wiese et al. 2016). The solution defines 4,551 equal-area 3-degree spherical cap mascon (mass concentration) elements directly from the intersatellite range-rate measurements within a Bayesian framework to remove correlated errors (Watkins et al. 2015). A coastal resolution improvement filter is subsequently applied to separate ocean and land mass from mascons that span coastlines (Wiese et al. 2016). Tidal alias terms (Ray and Luthcke 2006) were co-estimated along with the annual and semiannual components from the TWS time series. During the estimation process, a phase shift of 100 days was applied to each GRACE-FO alias term relative to those for GRACE in order to account for nodal plane differences. We used the formal errors that are distributed with the JPL mascon product, which represent the GRACE/FO measurement system error. We combined these in quadrature with leakage error associated with imperfections in the coastal resolution improvement filter by conservatively assuming the separation of land and ocean mass is in error by 50% (Wiese et al. 2016). Additional GRACE/FO data products (Save et al. 2016; Loomis et al. 2019) were used as noted below. Mascons in which ice cover exceeded 1% of the area were classified as “ice mascons" and were excluded from the TWS calculations (see Figure S2). Tests indicate that the ice coverage threshold (1%, 10%, etc.) does not affect the interannual variability as shown in Fig. 1; however, it does affect the trend. Thus, in an effort to be conservative in removing ice mass signal from the analysis, we used a 1% area cutoff to remove linear trends associated with ice mass loss.

In discussing the major droughts that have contributed to the step decrease in TWS, we refer to droughts and their durations and intensities as reported by Rodell and Li (2023). They applied a data clustering algorithm to the global, 2002–2021 GRACE/FO data record (Save et al. 2016; Save 2020) in order to identify spatially contiguous regions where the TWS anomaly exceeded one standard deviation from the mean seasonal cycle. The clusters of such data pixels were grouped temporally as well. This enabled automated, objective identification of droughts and pluvials (large scale and extreme wet events). These were then quantified using an intensity metric (Thomas et al. 2014) that represents the integral under the curve of non-seasonal TWS anomaly versus time, in units of km^3^ months (equivalently, GT∙months).

### Ocean Altimetry and Temperature

Global mean sea level changes were computed using the daily gridded product provided by the Copernicus Climate Change Service (C3S), version vDT2021 (Legeais et al. 2021). The effects of GIA and of subsidence due to present-day ice melting were removed from the GMSL time series with respective values of – 0.3 mm/yr (Peltier 2004) and – 0.13 mm/yr (Frederikse et al. 2017; Lickley et al. 2018). The drift of the wet troposphere correction of Jason-3 altimetry was corrected using the empirical estimate of Barnoud et al. (2023). The standard uncertainties associated with the GMSL time series were derived from the method and uncertainty budget detailed by Guérou et al. (2023). Note that the GIA model is different from the one used for the processing of the gravimetric data, but the uncertainties associated with the GIA correction are incorporated in the GMSL uncertainty budget.

Global mean thermosteric contributions to GMSL change can be estimated by subtracting satellite gravimetry measurements of BSL change (global mean ocean mass change converted to an equivalent height of water) from altimetry measurements of GMSL change or can be computed directly from temperature and salinity observations in the water column (e.g., Jayne et al. 2003). As the global mean halosteric sea level change due to salinity changes is supposed to be negligible (Gregory and Lowe 2000; Llovel et al. 2019), we only accounted for the global mean thermosteric sea level component. Only the local time average over 2005–2015 of the salinity measurements was used in the computation of the thermosteric component, avoiding any problem linked to salinity measurement drift observed after 2015 (Wong et al. 2020; Barnoud et al. 2021). Despite the fact that Argo floats only sample the top 2000 m of the ocean, multiple studies have demonstrated that the two estimates match reasonably well over the period between 2005 and roughly 2016 (Chambers et al. 2017; World Climate Research Programme Global Sea Level Budget Group 2018; Barnoud et al. 2021). Herein, the thermosteric component was computed as the mean of an ensemble of 10 in situ datasets based on temperature and salinity measurements from the Argo network (Argo 2023). The 10 datasets included four EN4.2.2 datasets provided by the Met Office Hadley Center (Good et al. 2013) with four different combinations of corrections applied for the expendable bathythermograph (XBT) and mechanical bathythermograph (MBT) data—XBT correction from Gouretski and Reseghetti (2010) and MBT correction from Gouretski and Cheng (2020), XBT and MBT corrections from Levitus et al. (2009), XBT correction from Cowley et al. (2013) and MBT correction from Levitus et al. (2009), and XBT correction from Cheng et al. (2014) and MBT correction from Gouretski and Cheng (2020), a dataset from the Institute of Atmospheric Physics from the Chinese Academy of Sciences (Cheng et al. 2017, 2020), the In Situ Analyse System (ISAS) 20 dataset (Gaillard et al. 2016), data from Ishii et al. (2017), the Grid Point Value of the Monthly Objective Analysis using the Argo data (MOAA GPV) version 2021 data (Hosoda et al. 2010), a dataset from the National Oceanic and Atmospheric Administration (Levitus et al. 2012; Garcia et al. 2019), and the Roemmich and Gilson (2009) data from the Scripps Institute of Oceanography. From these 10 datasets, the thermosteric sea level change of the ocean was computed up to 2000 m depth. The contribution of the deep ocean (below 2000 m depth) was added with a linear trend of 0.12 mm/yr (Chang et al. 2019). The standard deviation of the 10 members of the ensemble was used as a measure of uncertainty in the thermosteric sea level change data.

### Ocean Mass and Sea Level Budgets

The BSL was estimated over the ocean using the mean of three GRACE and GRACE-FO mascon solutions, from the Jet Propulsion Laboratory, the Center of Space Research (Save et al. 2016; Save 2020) and the Goddard Space Flight Center (Loomis et al. 2019). The ocean mass change was computed from the provided ocean bottom pressure data by removing the spatial mean of the so-called GAD product (Dobslaw et al. 2017) which accounts for the static atmospheric surface pressure (Chen et al. 2019). The effect of GIA was already removed from the mascon data using the ICE6G-D model (Peltier et al. 2018). The standard uncertainty of the BSL was conservatively estimated from the difference between the maximum and minimum values among the three solutions at each time.

Given BSL either from GRACE/FO or from altimetry less global mean thermosteric sea level, the contribution of TWS change to GMSL change can be computed by removing estimates of Greenland and Antarctica ice sheet mass losses, of other land glacier and ice cap ablation, and of atmosphere water vapor content variations. We corrected for the contribution of land ice mass changes using GRACE/FO data over glaciated regions (white areas in Figure S2). The monthly water vapor content variations of the atmosphere were computed from the European Centre for Medium-Range Forecasts atmospheric reanalysis version 5 (ERA5; Hersbach et al. 2020). A common mask was applied to altimetry, gravimetry, and Argo data to enable comparison. This mask excluded areas beyond ± 60° (lack of Argo data beyond ± 60° and of altimetry data beyond ± 66°), closed seas (lack of Argo data) and Indonesian seas, and coastal areas up to 200 km away from the coasts (lack of Argo data and issues of gravimetric signal leakage near the coasts). The common mask ensures comparison of the altimetry, gravimetry, and Argo data over areas covered by all three observing systems with good quality data.

### Satellite Laser Ranging

Prior to GRACE, space-based gravimetry was accomplished by satellite laser ranging (SLR) to mirrored, passive geodetic satellites, beginning with the launch of STARLETTE (*Satellite de taille adaptée avec réflecteurs laser pour les études de la terre*) in 1975 (Flechtner et al. 2021). After Stella launched in 1993, there were enough laser ranging satellites in orbit to derive mass change time series using the mascon approach (Rowlands et al. 2005) with sub-continental scale resolution. Herein, we use TWS mascon data (Figure S1) derived from SLR observations from 5–7 geodetic satellites; Laser Geodynamic Satellites 1 and 2 (LAGEOS-1, LAGEOS-2), STARLETTE, Stella, and Ajisai were available for the full span, and Larets and the Laser Relativity Satellite (LARES) were added to the solution when they became available in 2003 and 2012, respectively. We generated partial derivatives to spherical harmonic degree and order 10 and applied regularization following the same general procedures described by Rowlands et al. (2005). The solution time series was adaptively deseasonalized using the complete ensemble empirical mode decomposition with adaptive noise (Torres et al. 2011; Loomis and Luthcke 2014). Mass trends were calibrated to match GRACE/FO over a common span to mitigate impacts of parameter correlations (Loomis et al. 2019). This was further justified by results of our tests which demonstrated that the recovered SLR interannual signals are not dependent on the background gravity model, but that the recovered trends are.

### Model, Reanalysis, and Ancillary Data

TWS from hydrological models forced with atmospheric analysis and observation-based near-surface meteorology was used for comparison with and interpretation of the observed time series. The ISBA-CTRIP (Interaction Soil-Biosphere–Atmosphere, Total Runoff Integrating Pathways from the Centre National de Recherches Météorologiques) hydrological model provides an estimate of the climate-driven TWS variations until 2018 (Decharme et al. 2019). However, human-induced contributions to TWS changes (e.g., irrigation and other consumptive uses of surface and groundwater) have become significant over the last two decades (Rodell et al. 2018). Based on the WaterGAP Hydrological Model (Müller Schmied et al. 2021), Cáceres et al. (2020) estimated a human contribution of 0.37 (0.30 to 0.45) mm/yr to GMSL change over 2003–2016. To account for the TWS contribution to GMSL change, we summed the climate-driven contribution from the ISBA-CTRIP model and the 0.37 mm/yr sea level equivalent anthropogenic trend from WaterGAP.

The ERA5-Land reanalysis (Muñoz-Sabater et al. 2021) is a 9-km resolution simulation of the ERA5 land model with four soil layers totaling 289 cm depth, forced with hourly mean ERA5 10 m near-surface meteorology, incident long- and short-wave radiation, and precipitation (*P*). Monthly mean fields of resulting total evapotranspiration (ET), runoff (RO), *P*, and TWS were sourced through the Copernicus Climate Change Service (C3S), as were ERA5 (Muñoz-Sabater et al. 2021) sea-surface temperature (SST) and vertically integrated moisture flux divergence. ERA5-Land TWS was found by aggregating the volumetric water content of four soil layers and further adding snow water equivalent and vegetative water. Ancillary monthly mean data used to help interpret GRACE/FO TWS changes include the Global Precipitation Climatology Project, GPCP v3.2 *P* (Huffman et al. 2021); Global Land Evaporation Amsterdam Model, GLEAM v3.6a ET (Martens et al. 2017; https://www.gleam.eu/); the Multi-Forcing Observation-Based Global Runoff Reanalysis, G-RUN RO (Ghiggi et al. 2021; https://doi.org/10.6084/m9.figshare.12794075); and TWS from the WaterGAP 2.2d model (Müller Schmied et al. 2021) which was forced with observationally bias adjusted ERA5 data (Lange 2019; Cucchi et al. 2020). All data were interpolated to a 1.0° grid using the Grid Analysis and Display System (GrADS) analysis system.

## Results

### GRACE/FO TWS Variability

**Fig. 2:**
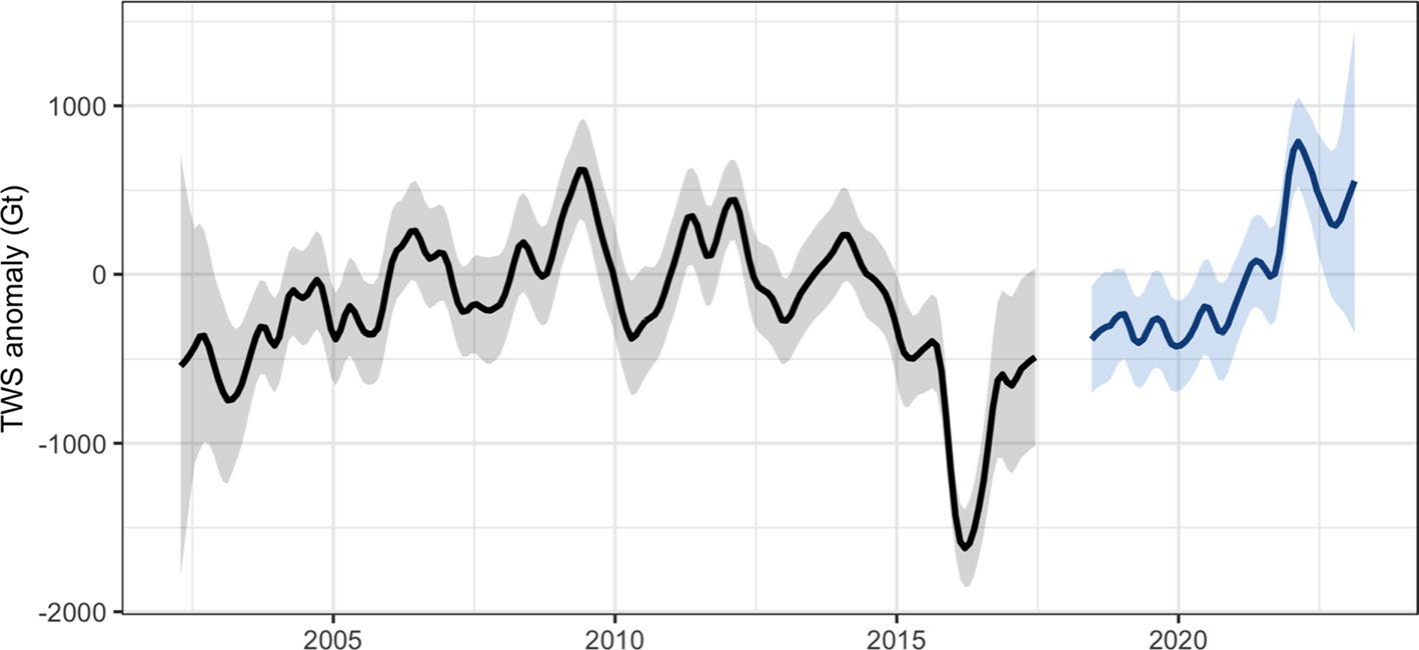
Anomalies (relative to the 2004–2010 mean) of terrestrial water storage (GT) from GRACE (black) and GRACE-FO (blue) averaged over the region of South America delineated in Fig. 3. The time series was deseasonalized and smoothed (7-month moving window with seasonal and trend decomposition using Loess). The shading indicates the formal errors

**Fig. 3:**
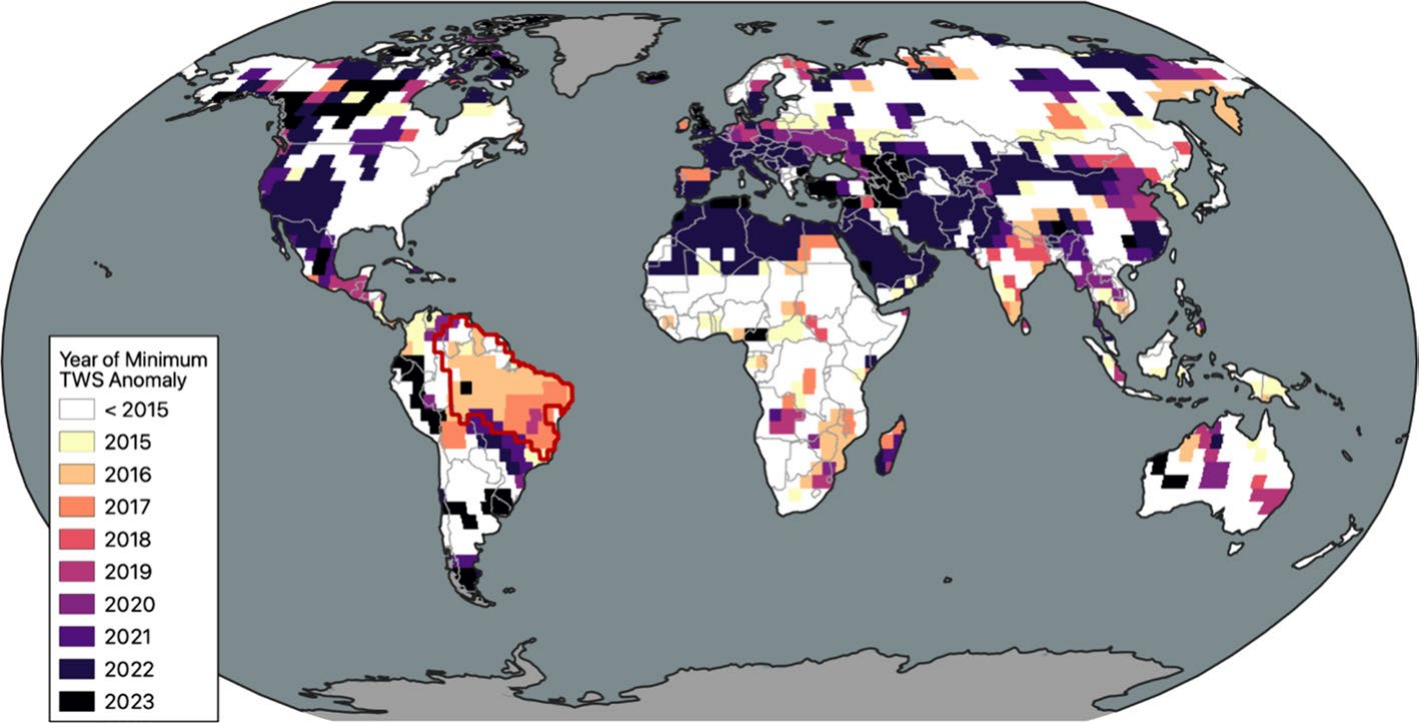
Map of mascons in which the GRACE/FO era minimum terrestrial water storage occurred after the start of 2015, color coded for the year of that minimum. The time series plotted in Fig. 2 is averaged over the South American region delineated in red

As shown in Fig. 1, there was a large, abrupt decline in TWS between May 2014 and March 2016, when the GRACE/FO era minimum occurred. During this period, the mean (deseasonalized) TWS decrease over non-ice land mass was approximately 22 mm. To determine if this decline represents a statistically significant structural change in the global land TWS time series, we used a Bayesian ensemble algorithm for changepoint detection (Zhao et al. 2019). This method quantifies the likelihood of detected shifts in the mean and trend by randomizing parameters and structure of time-series decomposition models, creating a posterior probability distribution from the ensemble, and arriving at a weighted average model using Bayesian model averaging. Stochastic sampling of the model space was implemented via Markov Chain Monte Carlo. Figure S3 shows the raw and deseasonalized mean global TWS GRACE/FO anomalies, along with examples of individual trend components from random model samples. From a complex model space of 60,000 Monte Carlo iterations, an abrupt (single month) change of – 3.2 mm was detected in January, 2015, with greater than 99.9% probability. Other possible changepoints were detected in April 2012 (– 1.9 mm) and December 2019 (+ 1.9 mm), but the 2015 decline represents the largest (mostly likely single) shift in global TWS in the GRACE/FO record. By repeating this test for all land mascons and mapping the probability of changepoint occurrence in 2015 (Figure S3b), we can determine the spatial origin of the abrupt decline. The proximal source was a drought in northeastern South America (delineated in Figs. 3 and S3b) that was the most intense dry event in the GRACE/FO data record (Rodell and Li 2023). Figure 2 plots a time series of TWS from that region. Concurrent droughts elsewhere in the world also contributed (Intergovernmental Panel On Climate Change 2023).

That global TWS has remained in a lower range since the initial decline can be attributed in large part to dry events in other areas of the world that followed the drought in South America. To illustrate, Fig. 3 indicates locations where the GRACE/FO era minimum TWS was recorded between January 2015 and May 2023. These locations encompass 52% of the global land excluding Greenland and Antarctica. All else being equal, the expected area percentage would be equivalent to the ratio of the number of 2015–2023 to 2002–2023 GRACE/FO monthly solutions, which is 37%. To determine the field significance of the 52% statistic, we applied a block bootstrap technique (Douglas et al. 2000), which maintains both temporal and spatial autocorrelations. This method involves permuting blocks of the available GRACE/FO months, retrieving mascon-level TWS time series at the corresponding sample times, and repeating the global test statistic for each iteration. Given a block length of 24 months (to capture autocorrelation from seasonal dependence) and a distribution of 500 bootstraps, the percent of non-ice land area reaching a post-2015 minimum is 52% or higher in only 3.6% of the bootstrapped samples. These tests strongly suggest that the 2014–2016 decline represents a statistically unusual, abrupt shift in global land TWS, while Figs. 2 and S3 implicate central Brazil as the primary source of the initial decline.

### Relationship between TWS and Sea Level

**Fig. 4:**
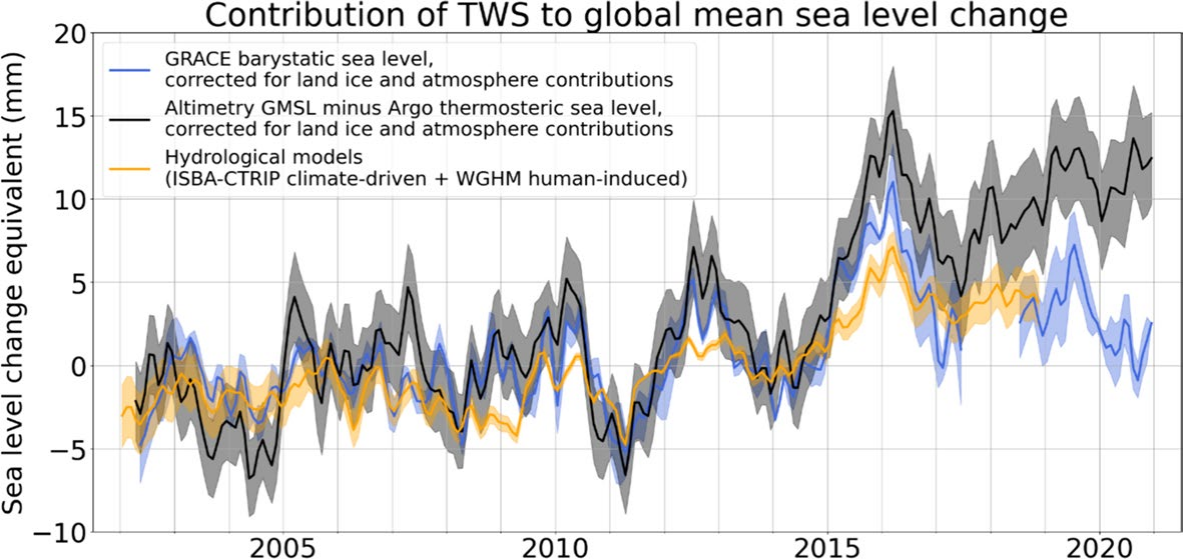
Time series of three estimates of the contribution of TWS to BSL (as sea level change equivalent in mm): (1) GRACE/FO observations of BSL after subtracting the contributions of glacier and ice sheet melt water inputs (based on Jet Propulsion Laboratory GRACE/FO data) and of atmospheric water vapor content variations (using the ERA5 reanalysis); (2) satellite altimetry measurements of GMSL after subtracting the contributions of melt water inputs, atmospheric water vapor content, and thermal expansion (based on Argo float data); and (3) hydrological model output (sum of the climate-driven contribution from ISBA-CTRIP model and human-induced linear contribution based on WaterGAP Hydrological Model, WGHM). Annual and semiannual signals have been removed. A mask is applied to gravimetry, altimetry, and Argo data over the ocean, excluding latitudes beyond ± 60°, closed seas, and Indonesian seas and coastal areas up to 200 km from the coastline

The effect of the decline in TWS on sea level is shown in Fig. 4, which compares the contributions of TWS to BSL as estimated using three different approaches. First, BSL was estimated from GRACE/FO observations over the ocean. Second, it was estimated from satellite altimetry measurements of GMSL after correcting for thermosteric effects using Argo float data. Land ice and atmospheric water vapor contributions were removed from both of these BSL time series. Third is a time series based on hydrological model output. While the two observation-based time series are in very good agreement during the first half of the period, typically remaining within 7 mm of each other and crossing frequently, they diverge after 2015 and end the study period about 10 mm apart, signifying an overestimate of the total sea level change from altimetry and/or an underestimate of its components from GRACE/FO or Argo. The hydrological model agrees well with GRACE/FO even beyond 2015, but with subdued extremes. Despite the discrepancies over the later years, both the GRACE/FO and altimetry-based estimates indicate the large rise in BSL between 2014 and 2016. The associated decline in TWS manifested as droughts in northeastern South America and elsewhere, which have been attributed to back-to-back El Niño events (Llovel et al. 2023), including the 2015–2016 “Extreme El Niño” (see Box 11.4 in Intergovernmental Panel On Climate Change (2023)).

### Historical Context

**Fig. 5:**
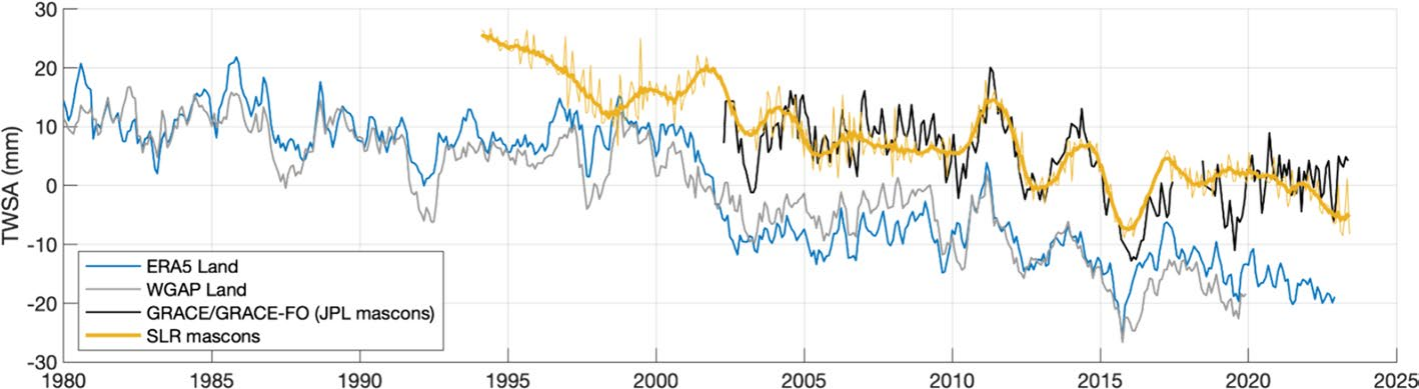
Deseasonalized time series of TWS anomalies (TWSA) from ERA5, WaterGAP, SLR, and GRACE/FO, excluding Greenland, Antarctica, and the gulf coast of Alaska region. Both unfiltered monthly SLR (thin gold line) and filtered (Complete Ensemble Empirical Mode Decomposition with Adaptive Noise) SLR data (thick gold line) are plotted. The SLR data represent the sum of mascons 1, 2, and 5–19 as shown in Figure S1. The model time series are plotted as TWS anomalies relative to a zero mean. The vertical offset of the observational time series is artificial and was inserted for visual clarity, as only the temporal variations are meaningful (the absolute anomaly values are not)

An important question is whether abrupt declines in TWS such as that during 2014–2016 (and perhaps abrupt gains) are unusual over the course of many decades. This question is difficult to answer using observations because global in situ measurements of the TWS components are woefully inadequate (Rodell and Reager 2023) while GRACE/FO has provided only two decades of observations to date. To provide historical context for the 2015 TWS decline, Fig. 5 compares time series from GRACE/FO (2002–2023), SLR (1994–2023), ERA5-Land (1980–2023), and WaterGAP (1980–2020). Reanalyses and global hydrologic models synthesize observational data with physically based constraints; thus, they may be instructive when investigating natural processes contributing to an abrupt decline in global TWS. During the concurrent period, 2002–2020, the four time series agree well, with correlation coefficients ranging from 0.70 (ERA5 vs. SLR) to 0.86 (WaterGAP vs. GRACE/FO).

**Fig. 6:**
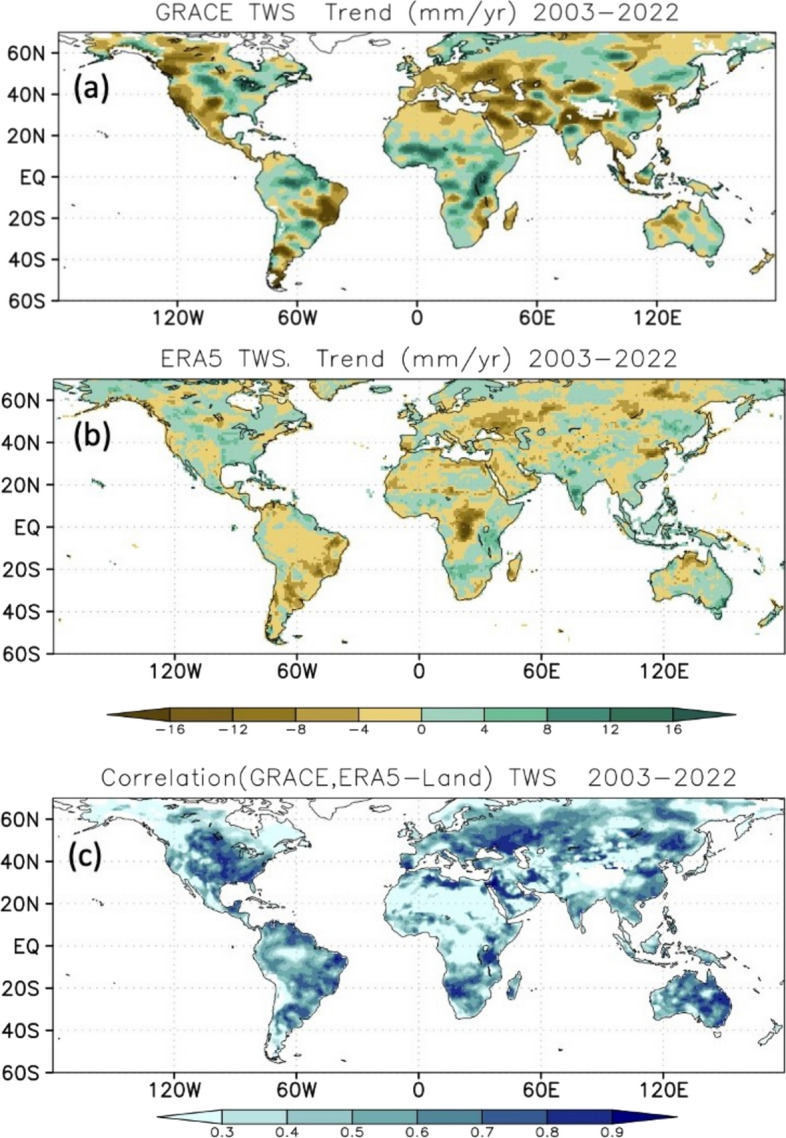
TWS trends (mm/yr) over the period 2003–2022 in **a** GRACE/FO and **b** ERA5-Land; and **c** correlations of monthly TWS anomalies between GRACE/FO and ERA5-Land. Ice sheets and areas of significant glacial extent have been masked (see Figure S2)

TWS’s abrupt decline around 2015 and its persistence in a lower range appears in all four time series. ERA5 displays a similar step decrease in 2002; however, it is not corroborated by the SLR or WaterGAP time series, which exhibit more gentle declines (with similar total magnitudes) between 1994 and 2005. To assess how unusual these events are in the context of interannual variability (distinct from long-term change), we first removed the linear trend and seasonal signals from each of the datasets and standardized the TWS anomalies to time series of Z-score statistics. We then identified TWS drying events with a Z-score < – 1 for at least 2 consecutive months. These events are ranked by duration and intensity in Figure S4. In all four datasets, the 2015 TWS decline was the strongest in terms of minimum Z-score. In all but ERA5, it also had the longest duration. Clearly, the 2014–2016 decline was unusual. The regional expression of the overall GRACE/FO TWS global trend and the ability of ERA5-Land to replicate this variability are displayed in Fig. 6. The confidence we have in the former is rooted in it being an observational product that has been evaluated and trusted for more than two decades (Humphrey et al. 2023; Rodell and Reager 2023). There is broad agreement between ERA5-Land and GRACE/FO in terms of monthly, regional TWS trend patterns over much of the globe (Fig. 6c). However, the pattern correlation between GRACE/FO and ERA5-Land trends is only 0.24 and ERA5 trends are roughly half those of GRACE/FO. Widespread losses across semi-arid to arid climates over Eurasia, the Middle East, and western North America constitute the major driver of the global downward GRACE/FO TWS trend. Though pattern agreement over Asia is fair, significant extraction of groundwater, river damming, and other human influences spanning the Middle East (Joodaki et al. 2014; Chao et al. 2018; Nikraftar et al. 2024) to north-west India (Rodell et al. 2009; Bhanja et al. 2020; Swain et al. 2022), and elsewhere (Rodell et al. 2018) have been documented. These effects are not modeled in ERA5-Land and could explain why certain regional TWS declines observed by GRACE/FO are substantially weaker or missing in ERA5-Land. Competing regions of significant TWS gain over the African Sahel and Rift Valley, eastern North America, Amazon basin, and many parts of Asia also exist. Notable discrepancies include the pronounced ERA5 drying in central Africa and the interior of the Amazon basin (Fig. 6b), which are opposite to those observed by GRACE/FO. These are areas that have few surface observations and are dominated by parameterized moist physics in the ERA5 atmospheric model. Reanalysis discrepancies that are likely related to advancements in satellite atmospheric temperature and moisture profiling in the assimilation data stream between 1998 and 2002 have been reported (Nogueira 2020; Hersbach et al. 2020). These improvements in observing capabilities continue during the GRACE/FO tenure (Hersbach et al. 2020). Ironically, improving the ability of atmospheric observations to offset assimilating model biases may have affected ERA5-Land TWS trends. There is also a junction in 2002 of the multiple streams in which the ERA5 and ERA5-Land reanalyses were produced.

Maps of coefficients of correlation between monthly GRACE/FO and ERA5-Land TWS anomalies (Fig. 6c) illustrate their degree of month-to-month agreement. Broad areas of high correlation include much of Eurasia and North America (r > 0.70), where observational networks are generally dense. In regions of sparse and variable rain gauge coverage, e.g., the Sahara, central Africa, and the interior of the Amazon, agreement is poor (Maidment et al. 2015; Nogueira 2020; Nicholson and Klotter 2021). Agreement in temporal variability (Fig. 6c.) is poor in regions of substantial seasonal snow cover or glacial extent (Alaska, western and eastern Canada, northern Siberia, and High Mountain Asia). In these regions, deficiencies in ERA5-Land physics (e.g., its lack of a glacier model (Muñoz-Sabater et al. 2021)), the sparseness of in situ and radiosonde observations for assimilation and calibration, and errors in the GRACE/FO TWS trends associated with GIA model uncertainty may contribute to lower time-series correlation (Hersbach et al. 2020; Mayer et al. 2021; Muñoz-Sabater et al. 2021).

**Fig. 7:**
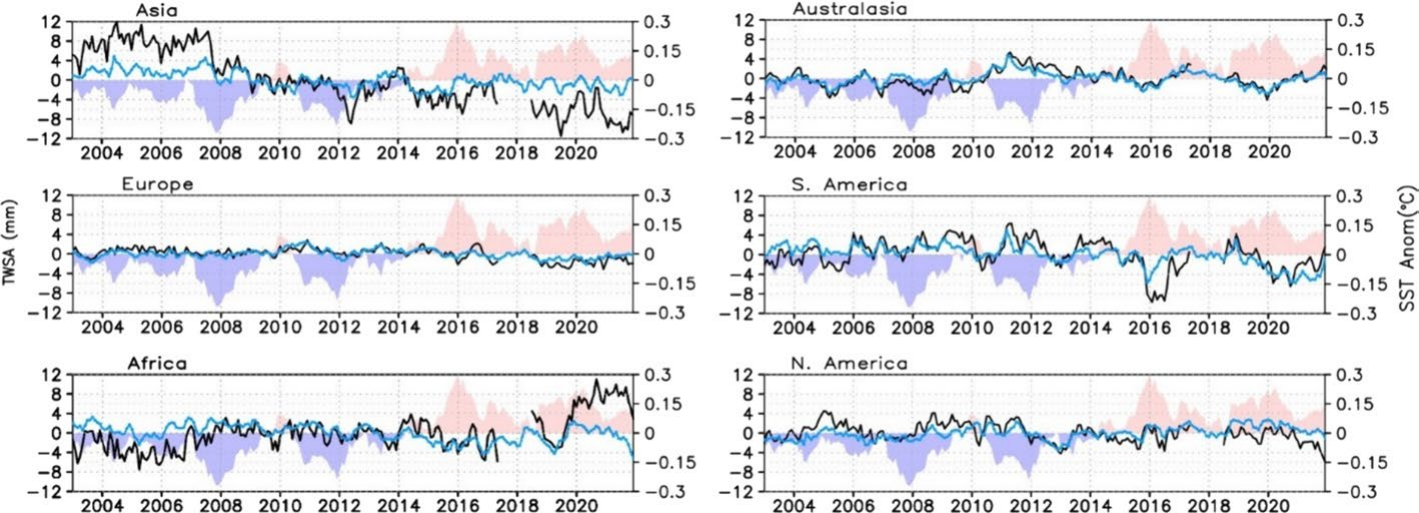
Deseasonalized time series of area-weighted TWS (mm, left Y-axis) over six near-continental regions based on GRACE/FO (black line) and ERA5-Land (blue line). The area-weighted scaling ensures that the sum of these contributions is the global TWS time series as shown in Fig. 5. Shading indicates global SST anomalies (°C, right Y-axis), relative to a 2003–2022 base period. See Figure S2 for definition of continental averaging domains

To quantify the larger scale importance of these spatial patterns to the global mean trends and the extent to which GRACE/FO and ERA5-Land agree, we calculated area-weighted (continent area over global land area, excluding the white areas in Figure S2) time series for six near-continental regions (Fig. 7). Also shown for each plot is the near-global mean SST anomaly time series. While no simple relationship exists between SST anomalies and TWS changes averaged at continental scales, the SST record does exhibit prominent interannual signals that have continental scale influence. Asia, as a whole, was the dominant contributor to declines in global mean TWS, with major droughts beyond the 2016 El Niño being crucial to sustaining low global mean TWS despite the upward trend present in many parts of Africa (according to the GRACE/FO data). We suspect that disparities between the magnitudes of ERA5-Land and GRACE/FO TWS variations and trends, most prominently over Asia, stem from two related factors. First, ERA5-Land does not simulate groundwater storage, thus limiting the range of variability of TWS. Second, human water management and consumption, especially groundwater withdrawals, are not simulated by the ERA5-Land system. In Europe, it also exhibited a downward TWS trend which had a much smaller weighted rate owing to its proportionally smaller area. Australasia and South America were largely responsible for the 2011 global TWS peak (Boening et al. 2012), with North America, Europe, and Africa also playing a role. South America experienced two large declines and recoveries in the post-2015 period, and the lack of a large positive TWS anomaly after 2015 is remarkable. South America also exhibited the largest swings from positive to negative TWS anomalies over the 2011–2016 period, but other continents, especially Asia, reflect this drawdown in TWS to varying degrees. South America is strongly influenced by ENSO and associated dislocations of moisture transport by the Walker circulation (Ropelewski and Halpert 1987; Castillo et al. 2014). After 2015, GRACE/FO and ERA5-Land agree that Asia and South America contributed most prominently to the diminished global TWS levels. The largest qualitative disparity between GRACE/FO and ERA5-Land TWS variations occurred in Africa, with GRACE/FO indicating continued accrual of water storage after a 2019 spike, while ERA5-Land TWS decreased substantially.

The emerging picture here is the dominance of Asia in sustaining a global, multi-decadal decline in TWS, largely driven by drought and exacerbated by human extraction of groundwater. A major additional contribution to the decline since 2015 comes from South America, whose hydrologic variations are large and strongly controlled by ENSO. In Asia, the drying trends are mostly found in the Middle East, Northern India, Northern China, and South-east Asia (Fig. 6). Partly counteracting those trends is a 20-year increase in TWS over Africa, which included the most intense extreme wet event (in central Africa) in the GRACE/FO data record (Rodell and Li 2023). Finally, as Fig. 6 shows, TWS variations within the continents are non-uniform, with consistency of trends being more the exception than the rule. Section 3.5 explores further the relationships between SST changes and TWS anomaly behavior at sub-continental scale.

### Water Fluxes and Moisture Transport

**Fig. 8:**
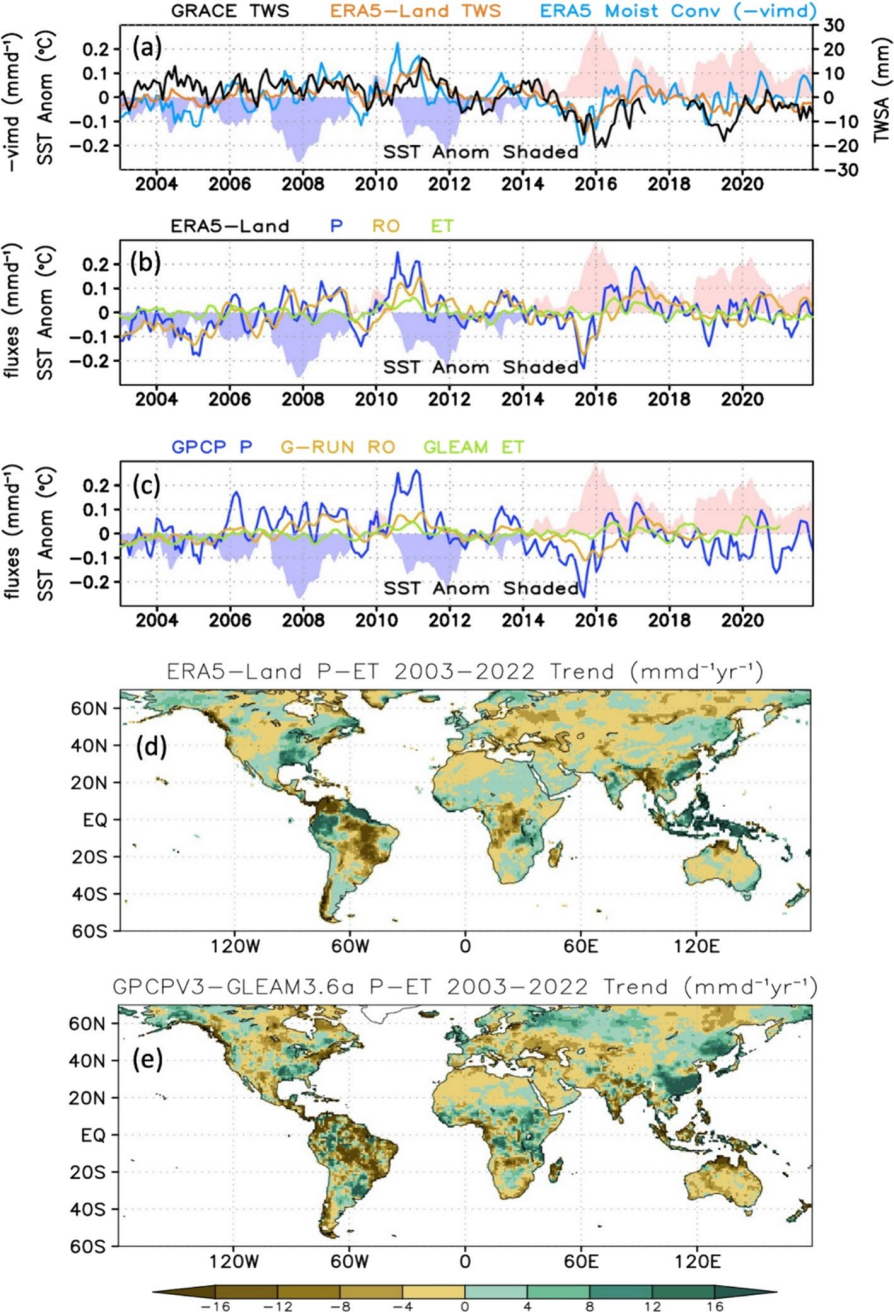
Comparison of ERA5-Land, GRACE/FO, and complementary observations: **a** deseasonalized time series of global land average (60S-70N) TWS anomalies (mm) from GRACE/FO, ERA5-Land, and ERA5 vertically integrated moisture flux convergence, **b** ERA5-Land precipitation (*P*), evapotranspiration (ET), and runoff (RO), **c** same as **b** but for GPCP *P*, GLEAM ET and G-RUN RO. Units in **b** and **c** are mm d^−1^, **d** 2003–2022 trend in ERA5-Land *P*-ET, **e** Same as **d** but for GPCP v3.2 *P*—GLEAM 3.6a ET. Units in **d** and **e** are mm d^−1^/yr. All anomalies are relative to the 2003–2022 monthly resolved climatological mean

In conjunction with a global SST rise of ~ 0.02 °C/yr during the GRACE/FO era, ERA5-Land TWS averages over 60S-70N (Fig. 8a) capture the increase and subsequent loss associated with the 2010/11 La Nina and 2015/2016 El Niño events. As noted above, ERA5-Land displays weaker TWS variability than GRACE/FO, especially over South America during the 2015/16 El Niño, and ERA5-Land TWS trends over the 2003–2022 period ( − 0.50 mm/yr) are also weaker relative to GRACE/FO (− 0.74 mm/yr). Although ERA5-Land TWS is an outcome of its water budget (*P*-ET-RO), a semi-independent and strong constraint on this budget is provided by the vertically integrated moisture flux convergence over the global land which has previously been considered a more robust estimate of *P*-ET than the directly computed diagnostics (Landerer et al. 2010; Trenberth et al. 2011). ERA5 vertically integrated moisture flux convergence is correlated with, and systematically leads, ERA5 TWS by 1 month (r = 0.41) due to the time scale of moisture import, precipitation, and hydrological response. ERA5 vertically integrated moisture flux convergence is anti-correlated with SST changes, owing to shifts in atmospheric circulation systems associated with internal climate oscillations such as ENSO and the Indian Ocean Dipole (IOD), which reduce precipitation over many tropical land areas during warm events (Ropelewski and Halpert 1987; Trenberth and Shea 2005; Trenberth et al. 2011; Bosilovich et al. 2020).

Figure 8b shows monthly anomalies of ERA5-Land *P*, ET, and RO, and Fig. 8c similarly compares observational estimates of the same from GPCP v3.2, GLEAM v3.6, and G-RUN RO. For both ERA5 and the observations, inputs of moisture from *P* are primarily balanced by losses through RO, although the response is relatively greater for ERA5 *P* and RO (r = 0.79) than for GPCP *P* and G-RUN RO (r = 0.64). For ERA5-Land, although ET is actually more strongly correlated with *P* (r = 0.88) than is RO, the magnitude of variations in ET is smaller. The GLEAM ET correlation with GPCP *P* is much lower (r = 0.22); yet, its comparison to ERA5 *P* (r = 0.34) improves considerably, which is logical because ERA5 *P* is an input to the water balance module used by the GLEAM ET algorithm and is also the forcing for ERA5-Land. The internal consistency between water and energy fluxes enforced by ERA5-Land model physics is typically lacking in observational estimates of fluxes that are derived separately from each other. The longer term variations in *P*, ET, and RO in ERA5-Land since 1980 display similar inter-relationships with ENSO variability (Figure S5) and suggest that a long-term global decline in TWS began prior to the GRACE period (Fig. 5). However, the ever-changing mix of observations assimilated by atmospheric reanalyses limits confidence in their ability to simulate long-term trends (Allan et al. 2020; Hersbach et al. 2020).

On monthly time scales, *P*-ET serves as a proxy for moisture flux convergence in the atmospheric water budget. Trends in *P*-ET during 2003–2022 from ERA5 and from GPCP v3.2 minus GLEAM 3.6a (Figs. 8d, e) show strong consistency with TWS trends from GRACE/FO and ERA5-Land (Fig. 6a, b). Pattern agreement with GRACE/FO over western Eurasia is particularly striking, signifying the important role of decreasing moisture transport into this region. GPCP and GLEAM also capture much of the implied role for moisture convergence that support GRACE/FO TWS increases over the Sahel and Rift Valley portions of Africa. However, correspondence between ERA5-Land *P*-ET and GRACE/FO TWS trends over South America is weak. ERA5-Land *P*-ET over central Africa, though mapping to its own TWS decreases there, is inconsistent with GRACE/FO measurements. Again, changes in satellite observations around the turn of the 21st Century likely caused time-dependent biases to propagate through the ERA5-Land hydrology.

In summary, ERA5 and observational estimates of TWS changes and their driving fluxes confirm both the substantial decline in TWS associated with the strong 2015/16 El Niño event and the longer term trend in TWS, as observed by GRACE/FO. These changes in TWS are clearly related to prior variations in moisture convergence over global land and the resulting precipitation deficits in South America, Eurasia, and Australasia, though ERA5-Land simulates TWS changes that are smaller in magnitude than those of GRACE/FO and fails to capture TWS patterns and trends over sparsely observed regions such as Africa.

### The Role of SST Changes

**Fig. 9:**
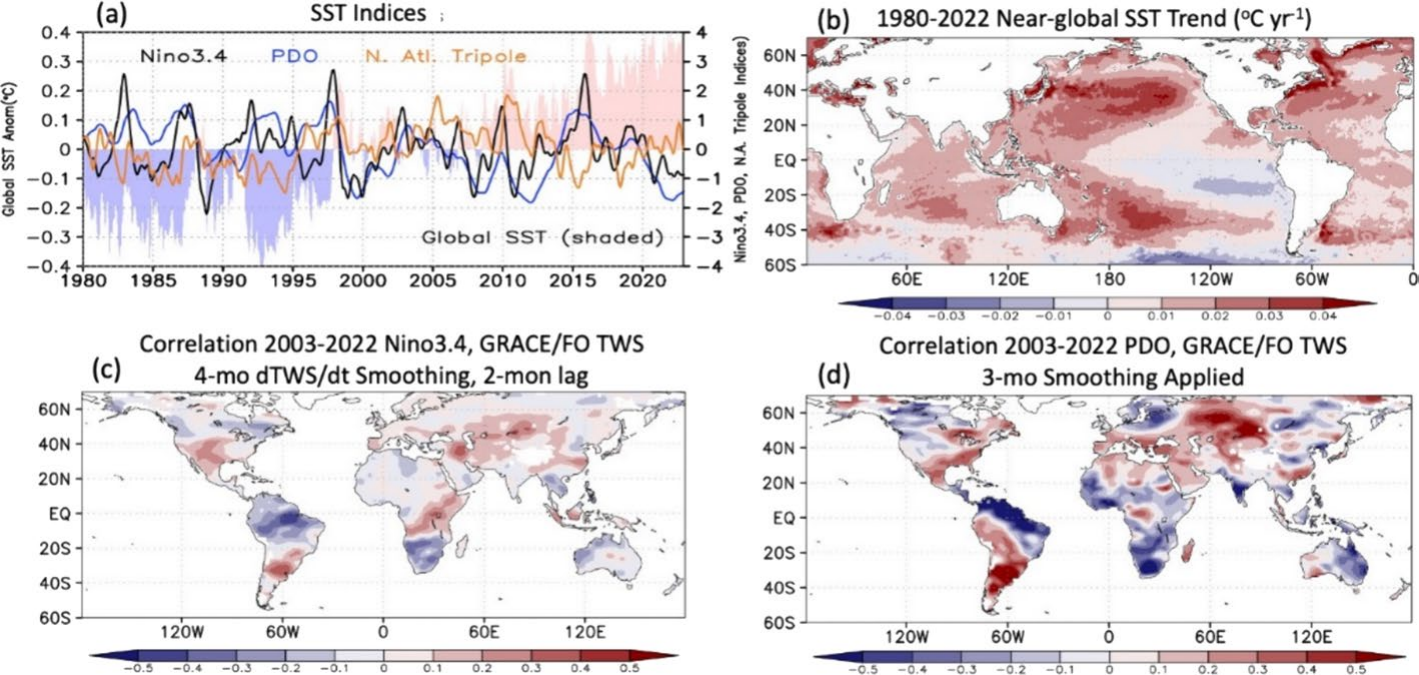
**a** Time series of near-global (60° S–70° N) mean SST anomalies, Niño 3.4 (black), the Pacific Decadal Oscillation (PDO) index with 1-year smoothing (blue), and a North Atlantic tripole index (brown). Negative PDO corresponds to anomalous positive SSTs in the central N. Pacific. **b** Near-global ERA5 SST trend, 1980–2022, °C/yr. **c** Correlation between the Niño 3.4 index and GRACE/FO d(TWS)/dt, the latter lagged 2 months with 4-month smoothing applied. **d** Correlation between the PDO index and GRACE/FO TWS, 3-month smoothing applied

ERA5-Land and observational flux estimates demonstrate the fundamental relationship between variations in atmospheric moisture delivery to land and TWS changes as observed by GRACE/FO (Fig. 8). We now explore how SST variability partially mediates this relationship (Fig. 9). Significant SST variations characterizing the 20-year GRACE/FO record (Fig. 9) include (1) six warm and eight cold ENSO events (https://www.ncei.noaa.gov/access/monitoring/enso/sst) and (2) sustained global SST increases exhibiting a striking near-global pattern of warming except over the eastern Pacific and extreme Southern Ocean. An empirical orthogonal function (EOF) analysis (not shown) reveals that ENSO-related interannual variations and secular trends explain the leading 29% and 11% of the monthly variance, respectively. Variations in other SST indices such as the Pacific Decadal Oscillation (Newman et al. 2016), an Atlantic tripole pattern (Czaja and Frankignoul 2002), and the IOD (Saji et al. 1999) are also presented. Each of these is known to have some dependence on tropical Pacific variability (Zanchettin et al. 2008; Newman et al. 2016; Ham et al. 2017; Casselman et al. 2021). While debate continues as to how anthropogenic warming versus natural climate variations constrain this evolving structure (Seager et al. 2019; Watanabe et al. 2021; Heede and Fedorov 2023), the observed pattern of warming differs from the more uniform increases simulated by coupled climate models (Wills et al. 2022; Duffy and O’Gorman 2023). This "pattern effect" has been shown to reduce amplifying cloud feedbacks relative to the models through altering lapse rates over the global tropics (Ceppi and Gregory 2019; Andrews et al. 2022). It has long been known that human-induced global warming leads to a strong land–ocean warming contrast, with greater warming over land and associated reductions in relative humidity (Sutton et al. 2007; Byrne and O’Gorman 2015; Seneviratne et al. 2016; Wainwright et al. 2021; Intergovernmental Panel On Climate Change 2023).^1^ But how this “pattern warming” is connected to regional trends in TWS (Fig. 6a, b) and the role of various processes needs further study.

Potential responses to SST pattern anomalies are not confined to long-term, secular trends but can also operate on interannual time scales (Izumo et al. 2019; Ceppi and Fueglistaler 2021). On the other hand, changes in land and ocean temperatures can be partially decoupled, such as during the so-called “hiatus” period of the early 2000s (Seneviratne et al. 2014), showing that SST changes do not necessarily affect land responses, in particular in the context of greenhouse gas forcing. In addition, any SST-induced adjustments over land are not uniformly distributed given regional hydrologic anomalies noted in previous studies (Hoerling and Kumar 2003; Lyon and DeWitt 2012; Schubert et al. 2016). Evidence for the influence of ENSO during the GRACE/FO era is seen particularly in correlations of d(TWS)/dt variations over South America, the southwestern U.S., southern and eastern Africa, and in the wave-like structure across northern Asia (Fig. 9c). Correlating SST anomalies with *P*-ET from GPCP and GLEAM ET (not shown) reveals similar patterns with even stronger correlations. The weaker GRACE/FO d(TWS)/dt correlations likely reflect the additional influence of RO on d(TWS)/dt behavior. Modes of tropical SST variability in other ocean basins (e.g., the Atlantic El Niño) also can assume importance in effecting TWS changes. For example, the IOD (Saji et al. 1999) can modulate rainfall over much of eastern Africa. A strong relationship (r = 0.66) between that mode and d(TWS)/dt over eastern Africa during 2003–2022 (Figure S6) exceeds that produced by ENSO (Fig. 9c).

Poleward propagating waves from ENSO events are also an important forcing for the Pacific Decadal Oscillation (Newman et al. 2016) as evidenced by their phasing in Fig. 9a (r = 0.53 with Niño 3.4 leading by 2 months). In Fig. 9d, the alternating pattern of correlation between the Pacific Decadal Oscillation index and GRACE/FO TWS extending across central Asia suggests modulation of storm tracks and their associated hydrologic processes. This pattern is replicated by replacing GPCP v3.2 *P* with TWS (not shown). The Pacific Decadal Oscillation, integrating the atmospheric forcing of SST, can be a marker of teleconnections extending around the higher latitudes. Likewise, TWS integrates *P*, ET, and RO anomalies in patterns reflecting the wave trains spanning the high latitudes.

Though direct greenhouse gas forcing and SST-induced anomalous moisture transport between land and ocean are essential in modulating TWS changes, it must be stressed that unforced climate system variability remains a major seasonal to interannual determinant of atmospheric circulation, weather, and, hence, hydrologic behavior. The influence of internal climate variability convolved with the preconditioned hydrologic state can often dominate (Miralles et al. 2019; Wehrli et al. 2019). A further cautionary note is that relationships between SST patterns and hydrologic response can be non-stationary (Torralba et al. 2015; Martija-Díez et al. 2023). More generally, interbasin SST connections via the atmosphere ensure that SST indices or modes are not independent (Cai et al. 2019), adding complexity to hydrologic responses and TWS evolution over land.

## Discussion and Conclusions

The global mean variations in TWS can be examined at the continental level (Fig. 7) and also related to major droughts and pluvials. Referring to extreme TWS events in the 2002–2021 GRACE/FO data identified by Rodell and Li (2023), the 2014–2016 abrupt decline in global TWS was kicked-off by a drought in northern and central Brazil (intensity of – 10,513 GT months during August 2015 to January 2017) that was the most intense in the GRACE/FO record and dominated the TWS signal for South America in its entirety (Figs. 2, 7). In total, 13 of the 30 most intense droughts occurred during or after the 2014–2016 global TWS decline (their location, timing, and intensity are illustrated in Figure S7), helping to suppress global TWS since that time. Rodell and Li (2023) identified two sub-continental regions (sub-Saharan Africa and west central South America) with coherent tendencies of dry events being more common than wet events in the first half of the 2002–2021 study period and the opposite in the second half of the period, suggesting a tendency toward wetting in those regions since 2002. On the other hand, there were three regions of coherence (southwestern North America, south-eastern Brazil, and a large swath from southern Europe across the Middle East and Arabian Peninsula to south-western China and Bangladesh) where the frequency of wet events notably decreased while dry events dominated during the second half of the study period. They noted both consistencies and inconsistencies between these tendencies and IPCC AR6 model predictions of precipitation change (Intergovernmental Panel On Climate Change 2023). Zonal mean TWS from GRACE/FO has been increasing between 5°S and 15°N and decreasing in the 10°–20° S and 25°–45° N bands (Dunn et al. 2024). There is much debate and little consensus about how patterns of wetting and drying will manifest in a warming world (Zaitchik et al. 2023); hence, it is difficult to evaluate whether the observed patterns are consistent with predictions and likely to persist.

Returning to Fig. 5 and the question of whether the 2014–2016 decline in global TWS was unusual, we evaluate three factors: magnitude of change (≤ − 15 mm), slope of decline (≤ − 1.0 mm/month), and whether there was a sustained recovery toward the pre-decline time series mean. As observed by GRACE, TWS decreased by 23 mm between May 2014 and March 2016 (22 months; slope = − 1.0 mm/month). A decline of 22 mm between April 2011 and February 2013 (22 months; − 1.0 mm/month) was nearly as large and steep. A third decline, 17 mm between May 2002 and March 2003 (10 months; − 1.7 mm/month), was the only other in the GRACE/FO record to meet the magnitude and slope criteria. Following the 2011–2013 decline, TWS recovered to exceed the pre-decline mean 11 months later, during January–March 2014. Following the 2002–2003 decline, TWS exceeded the pre-decline mean 13 months later, in April 2004, and stayed above that level for a year. However, since the 2014–2016 decline ended, TWS has remained more than 1 mm below the pre-event mean in every month except September 2020 (54 months later), when it briefly spiked upward. Based on these three criteria as well as the statistical analysis presented in Sect. 3.1, we conclude that the 2014–2016 TWS decline was unique in the 2002–2023 GRACE/FO data record. As for the ERA5-Land and WaterGAP time series, there is agreement with GRACE regarding the depth and steepness of the 2011–2013 and 2014–2016 declines, as well as the recovery following the first and lack of recovery following the second. Prior to the GRACE/FO period, both models indicated steep declines that began in 1985, 1991, and 1997. However, none of these (in the case of either model) met both the magnitude and slope criteria, and all were followed within 1–2 years by sharp recoveries to above the pre-decline means. Of principal interest is a 22 mm decline in TWS indicated by ERA5-Land that began in November 2000 and reached a minimum in September 2002 (20 months; − 1.1 mm/month), following which TWS never recovered to the pre-decline mean. The real world occurrence of such a decline in global TWS would invalidate our conjecture that the 2014–2016 TWS decline was unprecedented in the past 43 years. However, while both the WaterGAP and the SLR time series indicate that a decline in TWS occurred during that approximate timeframe (2000–2003) which met the magnitude and non-recovery criteria, both suggest it had a gentler slope, failing the ≤ − 1.0 mm/month test. WaterGAP indicates a decline of 17 mm over July 2000 to April 2003 (-0.5 mm/month). SLR indicates a decline of 11 mm (less than the 15 mm threshold) over January 2002 to January 2003 (− 0.9 mm/month). We are inclined to trust the SLR and WaterGAP data over ERA5-Land, and hence to conclude that the 2014–2016 decline in global TWS was, indeed, unprecedented during the past 4 decades. Our reasoning is twofold, First, if we break the period of Fig. 5 into three epochs, 1994–2001, 2003–2014, and 2015–2020, next average each of the four time series over those epochs, and finally compare the epoch-to-epoch changes, there is general consistency among all except ERA5 (Table 1), which has a steeper slope prior to 2003 and gentler slope after 2003 compared with the other time series. Second, as mentioned before, the multitude of consequential changes in meteorological observing systems between 1998 and 2002 that provided data to be assimilated by ERA5 during those years likely produced temporal discontinuities (Hersbach et al. 2020) which contributed to spurious precipitation trends (Nogueira 2020; Allan et al. 2020; Gleixner et al. 2020), thus casting doubt on any unusual events that occurred during that period, including ERA5-Land’s November 2000 to September 2002 TWS decline. More generally, (Scanlon et al. 2018) called into question the ability of global models to represent large scale, multi-annual TWS variability.

**Table 1:** Changes in global mean TWS (mm) between three epochs, 1994–2003, 2003–2014, and 2015–2020, based on GRACE/FO, SLR, ERA5, and WaterGAP

Dataset	dTWS (mm) [2003–2014] minus [1994–2003]	dTWS (mm) [2015–2020] minus [2003–2014]
GRACE/FO	N/A	− 9.1
SLR	− 11.0	− 7.4
ERA5	− 17.2	− 5.5
WaterGAP	− 11.5	− 11.9

While reanalyses and models are imperfect, they are helpful when attempting to infer physical mechanisms for observed phenomena. During the GRACE/FO era, global mean ocean temperature rose at a rate of 0.21 °C per decade, continuing, since 1980, a series of largest decadal scale trends seen since 1900 (see Fig. S3 in Xu et al. (2022); also https://www.ncei.noaa.gov/access/monitoring/climate-at-a-glance/global/time-series). At the largest scale, these warmer ocean temperatures cause more atmospheric upward motion and precipitation over the oceans, while in general, the opposite is true over land (Fasullo 2010; Lambert et al. 2011). On the other hand, land temperatures have also continued to rise rapidly and faster than those in the ocean, which is therefore unable to supply enough moisture to sustain relative humidity levels, leading to enhanced drying of the surface through evaporation (Sutton et al. 2007; Byrne and O’Gorman 2015; Intergovernmental Panel On Climate Change 2023). The resulting large-scale changes in vertically integrated moisture flux convergence and *P*-ET (shown in Fig. 8) in turn would be expected to drive declines in TWS, as observed by GRACE/FO (Figs. 6, 7). This leads to the supposition that a long-term downward trend in TWS is ultimately being driven by global warming. If true, the decline would not necessarily be gradual (as exemplified by recent global mean annual temperatures) and could instead manifest as an abrupt change like the one that occurred during 2014–2016. Variability internal to the climate system on interannual and longer scales (e.g., ENSO, PDO, IOD, and the Atlantic Meridional Oscillation) all exert significant influences on TWS changes. Underlying the global mean decline in TWS is a preponderance of extreme hydrological events since 2015 (Rodell and Li 2023), with the balance of those events being more dry than wet. At the continental scale, only Africa is currently resisting that trend (Fig. 7). The warmest 9 years in the modern global temperature record all have occurred since 2015, with 2024 certain to make it 10. Sudden TWS shifts can be expected to be convolved with a secular decline stemming from the erratic pace of warming.

Conclusively explaining the post-2015 divergence between GRACE/FO and altimetry minus thermosteric estimates of BSL will require further work. There are reasons to be uncertain about all three of the key observational inputs. Barnoud et al. (2023) discussed updates to the wet troposphere corrections for the Jason 2/3 altimeter time series that explain part of the gap. Argo profiling float measurements have been shown to contain spurious signals that can lead to biases in global mean thermosteric estimates when the salinity data after ~ 2015 are included (Hakuba et al. 2021). While we do not use the Argo salinity data for the global mean thermosteric sea level estimate here, undersampling the energetic ocean (i.e., eddies) can also introduce biases in the global mean thermosteric sea level variations (Lyman and Johnson 2023). GRACE/FO instrument measurements are in principle not prone to bias or drift, but the uncertainty levels of the monthly mass change observations vary over time, depending on ground-track coverage, thermal stability of the satellites, and external space-environmental factors (e.g., solar activity). Various studies have documented bias-free consistency of the GRACE and GRACE-FO data records, despite the 11-month gap between the missions (Landerer et al. 2020; Velicogna et al. 2020). To isolate surface mass (e.g., TWS or BSL) trends in the GRACE/FO observations, geophysical corrections are required to account for ongoing GIA; GRACE/FO uses as a standard the model of Peltier et al. (2015). Differences across an ensemble of GIA models equate to BSL trends of up to 0.5 mm/yr (Meyssignac et al. 2019). However, considering only state-of-the-art, global GIA models (such as Peltier et al. (2015), the actual resultant BSL trend uncertainty is estimated to be less than 0.2 mm/yr (Caron et al. 2018). Errors in accounting for GIA manifest as a constant bias in the GRACE/FO based TWS and BSL trend estimates, but they do not affect year-to-year variations. Ocean altimetry data are similarly corrected for GIA, with errors therein affecting sea-surface elevation change estimates in the same direction as they affect BSL change estimates from GRACE/FO. The two BSL time series (one from GRACE/FO, the other from altimetry-Argo; Fig. 4) are in good agreement prior to 2015 and diverge rapidly thereafter. This does not prove their accuracy prior to 2015, but considering that errors in the GRACE/FO based BSL time series would likely be steady (i.e., appear as a secular drift), the pre-2015 agreement and post-2015 divergence suggest an issue in the altimetry and/or Argo data as the root cause of that divergence.

As the planet continues to warm and ENSO cycles through its phases, it will be interesting to see if TWS will rebound to pre-2015 values, hold steady, or perhaps resume its decline. Future continuity (or enhancement) of the current suite of global sea level and terrestrial hydrological observations will be crucial for quantifying water cycle consequences of climate change, including long-term trends and shifts in seasonality and interannual variability, while constraining observing system biases and uncertainties.

## Supplementary Information

Below is the link to the electronic supplementary material.

Supplementary file1 (DOCX 1664 kb)

## Data Availability

GRACE/GRACE-FO data are available at http://grace.jpl.nasa.gov. The C3S altimetry data are available at https://doi.org/10.24381/cds.4c328c78. The Argo data were collected and made freely available by the International Argo Program and the national programmes that contribute to it (https://argo.ucsd.edu, https://www.ocean-ops.org). The Argo Program is part of the Global Ocean Observing System.

## Footnotes

1 To maintain planetary radiative equilibrium in the face of increasing SSTs, atmospheric profiles stabilized by deep moist convection (lapse rate reductions) over ocean propagate to near-global extent, increasing energy transport to land. To equilibrate, the atmosphere/land system must lose energy radiatively which induces subsidence, inhibiting moisture import. The smaller land surface water content compared to the ocean surface means that evaporation becomes less effective as a cooling agent and land surface temperatures must elevate (contributing to energy loss to space; sensible heat loss becomes much more important than over ocean).
