# Supplementary material for: An Abrupt Decline in Global Terrestrial Water Storage and Its Relationship with Sea Level Change
Source: Surv Geophys. 2024 Nov 4;45(6):1875–902. doi: 10.1007/s10712-024-09860-w (PMC11671563; doi:10.1007/s10712-024-09860-w)
Supplement: Supplementary file 1 — Supplementary file1 (DOCX 1664 kb) [file 10712_2024_9860_MOESM1_ESM.docx]

Supplementary Information


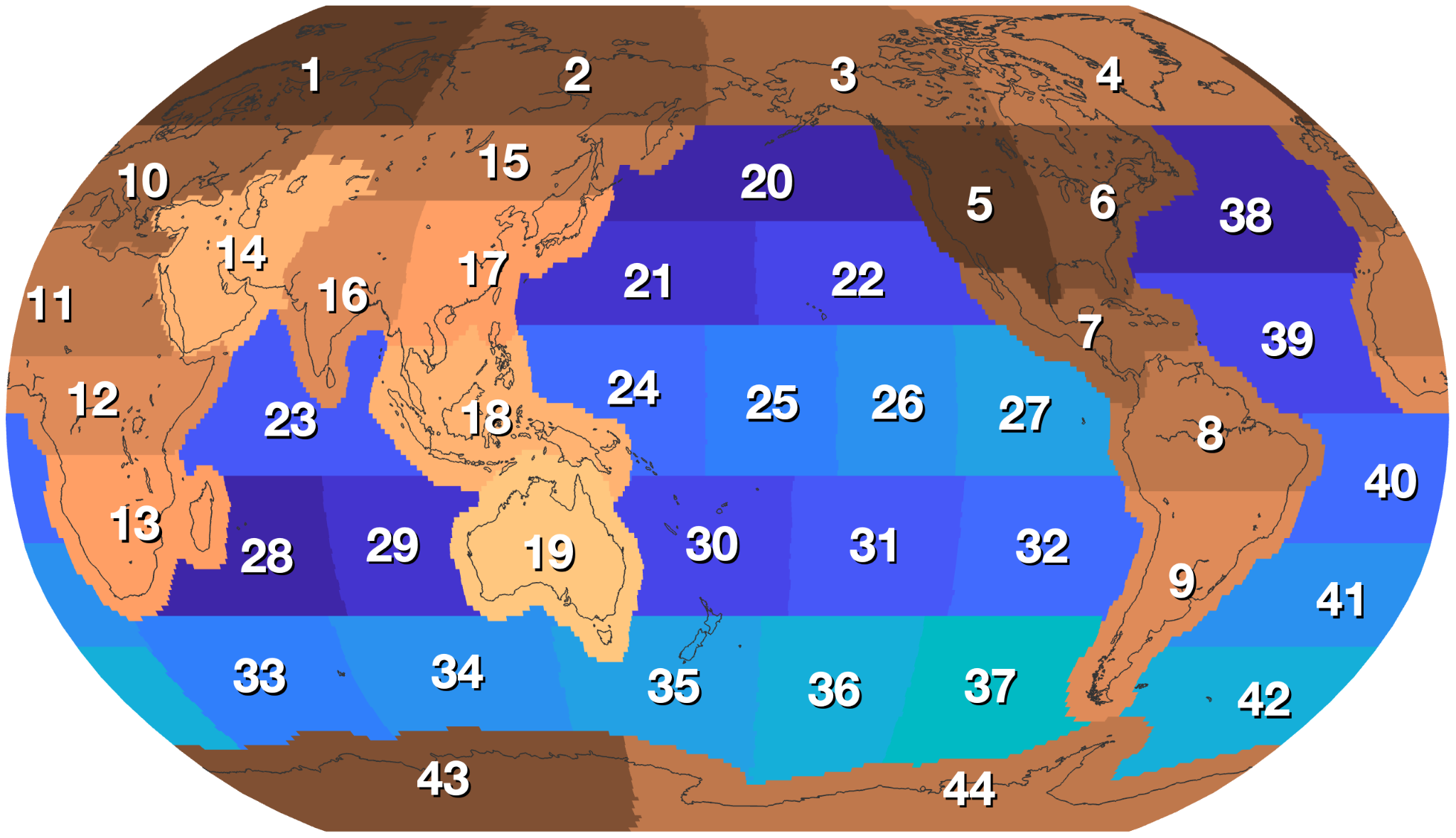


Figure S1. Locations of mascons used in deriving TWS (and ocean mass) time series from SLR data.


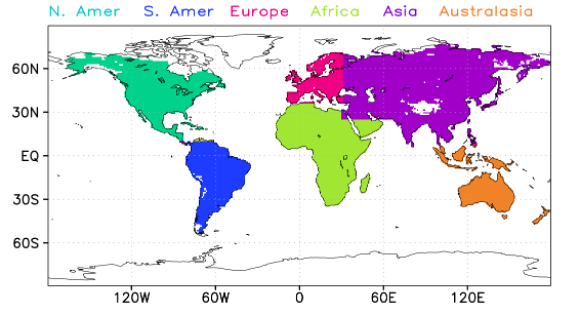


Figure S2. Continental averaging domains are color coded. Note that “Africa” and “Asia” each include part of the Arabian Peninsula. White indicates glaciated regions that were excluded when computing TWS anomalies and other regional averages.


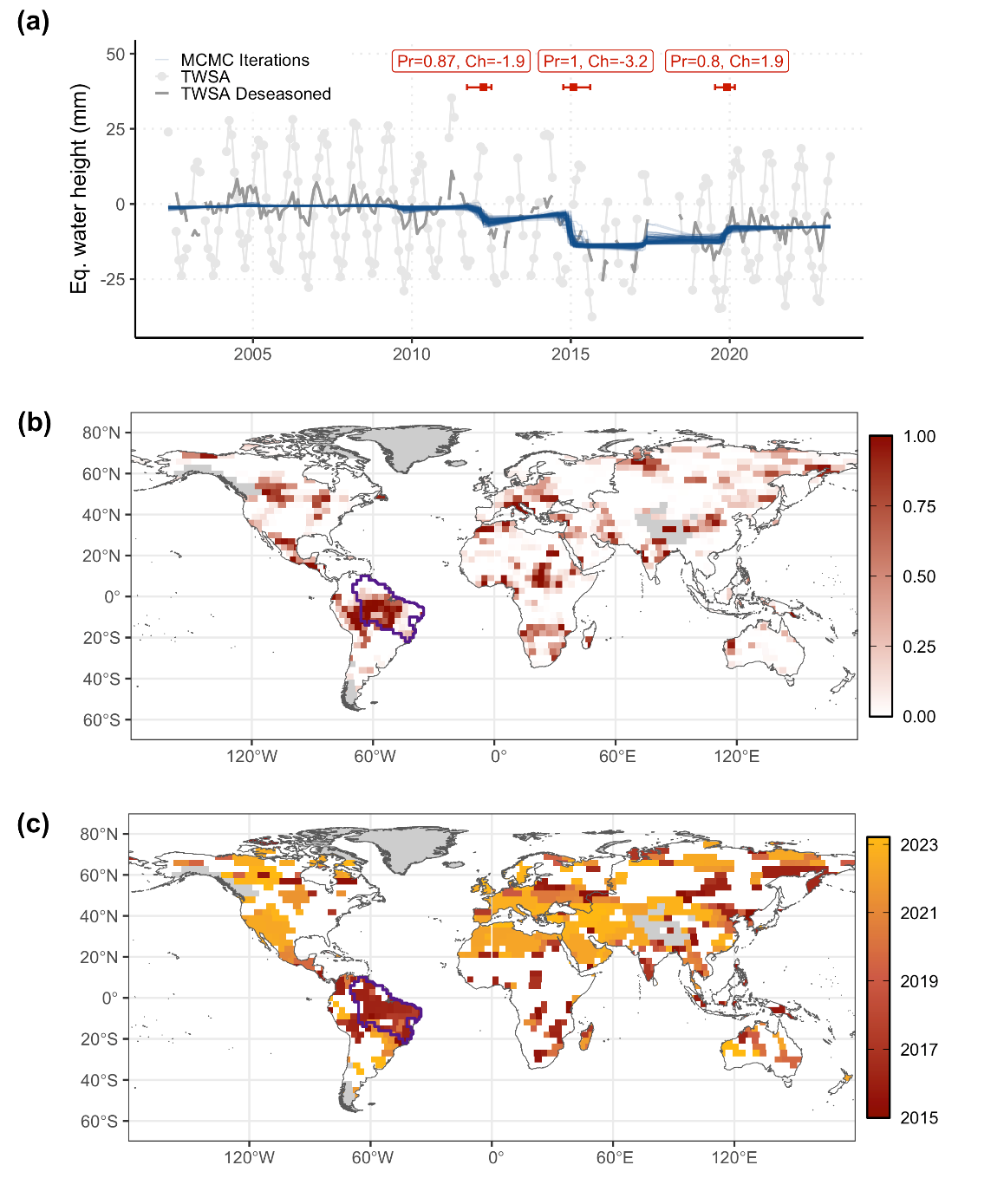


Figure S3. a) Timeseries of raw and deseasonalized TWS equivalent water height, along with 100 iterations of a Bayesian changepoint detection algorithm. Red annotations show the months corresponding to the three most likely changepoints from an ensemble of 60,000 models, along with their credible intervals, probability (Pr), and total single-month change (Ch, mm). b) The cumulative probability of a changepoint occurring during 2015 at each mascon, using a 60,000 model ensemble for Bayesian inference of mascon-level deseasonalized TWS. c) The year of absolute minimum in TWS, after removal of annual and semi-annual signals. Minima occurring prior to 2015 are masked in white. Land mass with significant ice cover is masked in grey. The delineated region in South America is that over which the time series in Figure 2 was averaged.


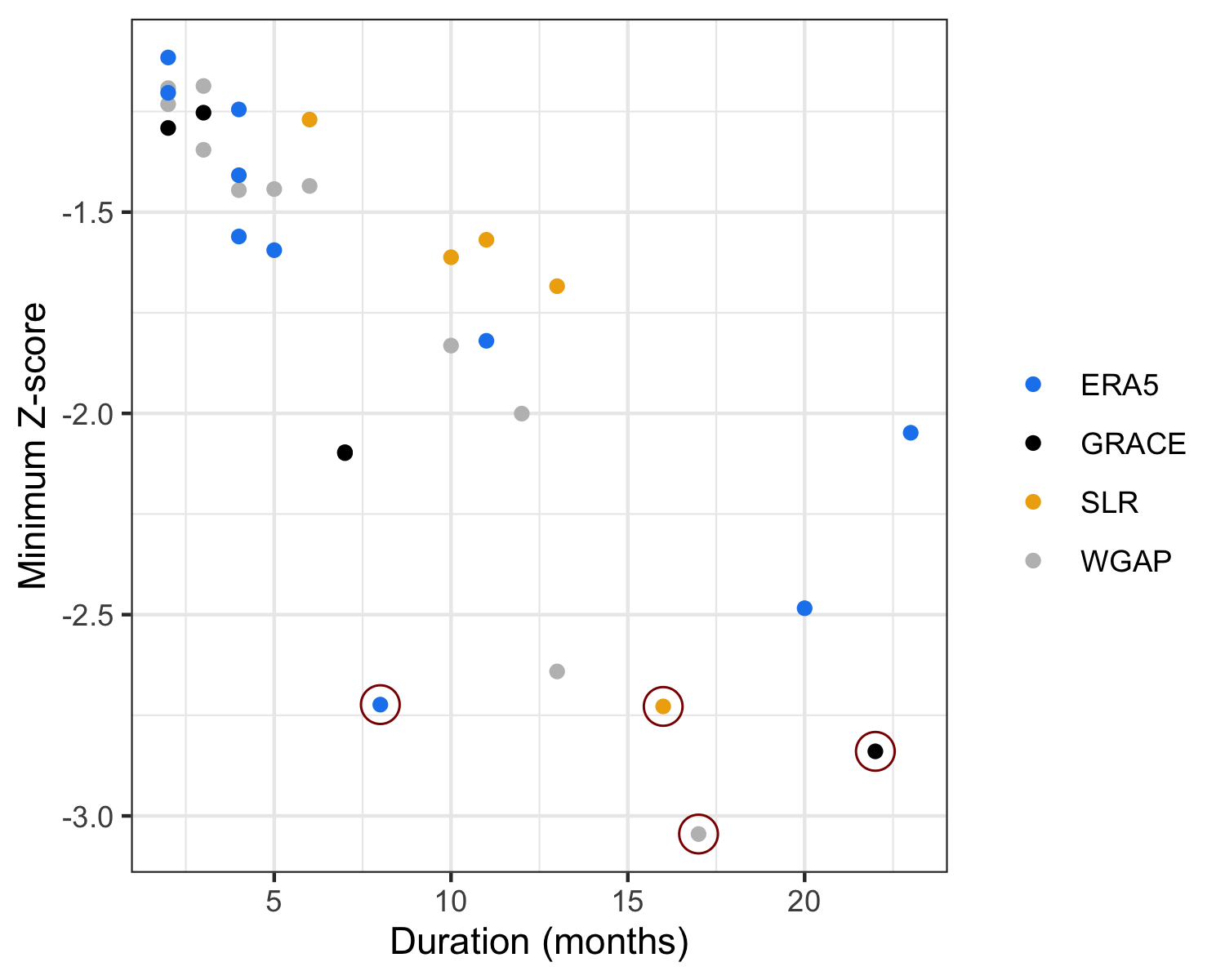


Figure S4. Global drying events with a mean absolute Z-score < -1, ranked by duration (x-axis) and minimum Z-score (y-axis) for observational (SLR and GRACE/FO) and model (ERA5 and WGAP) datasets. The 2014-2016 TWS decline is circled in red for all four datasets. Note that the length of record and standard deviation vary between datasets.


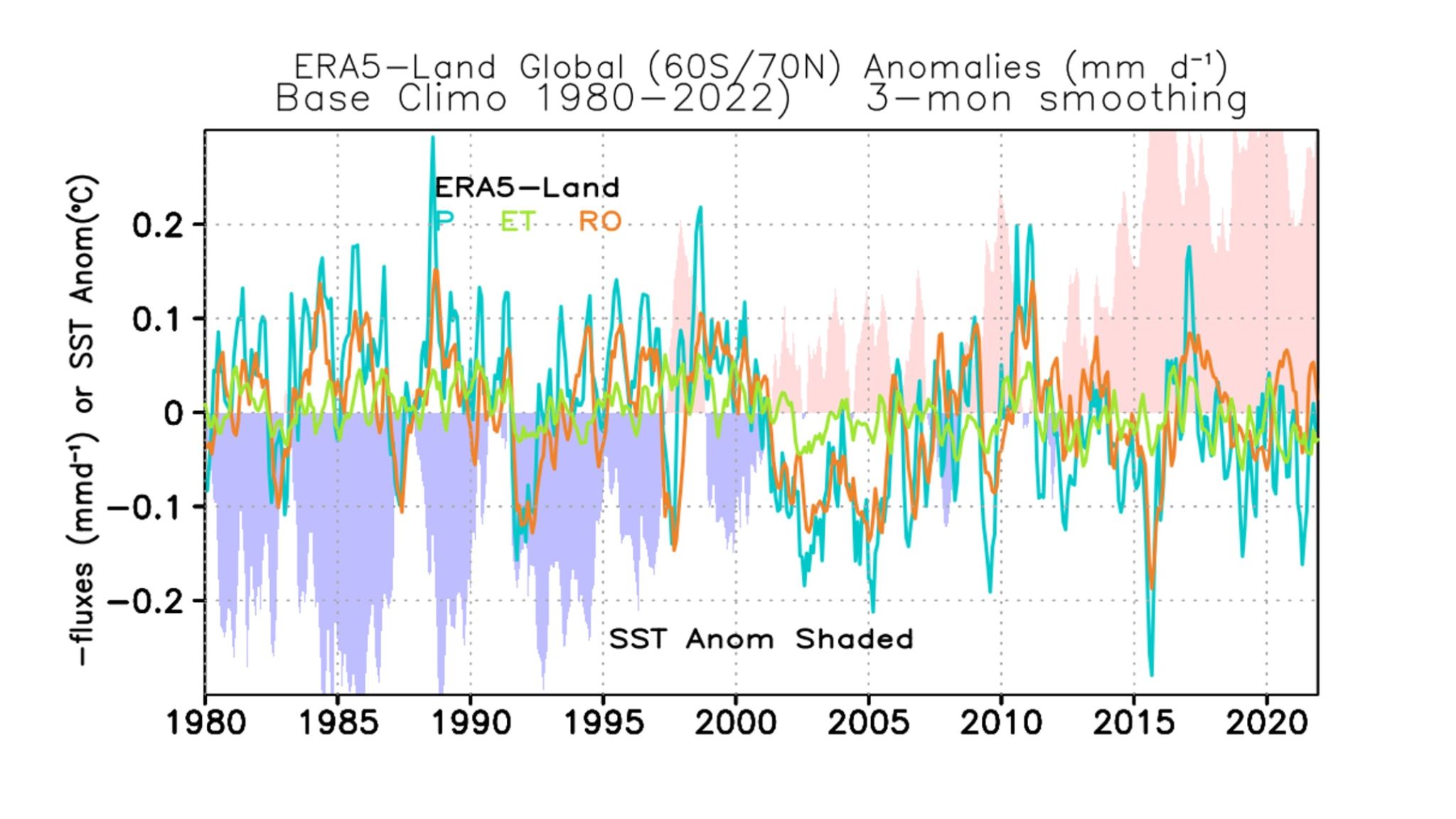


Figure S5. (a) Time series of ERA5-Land global land anomalies of *P*, ET and RO. Anomalies of fluxes and global mean SSTs are with respect to a 1980-2022 monthly climatology. Units are mm/day. A 3-month smoothing window has been applied.


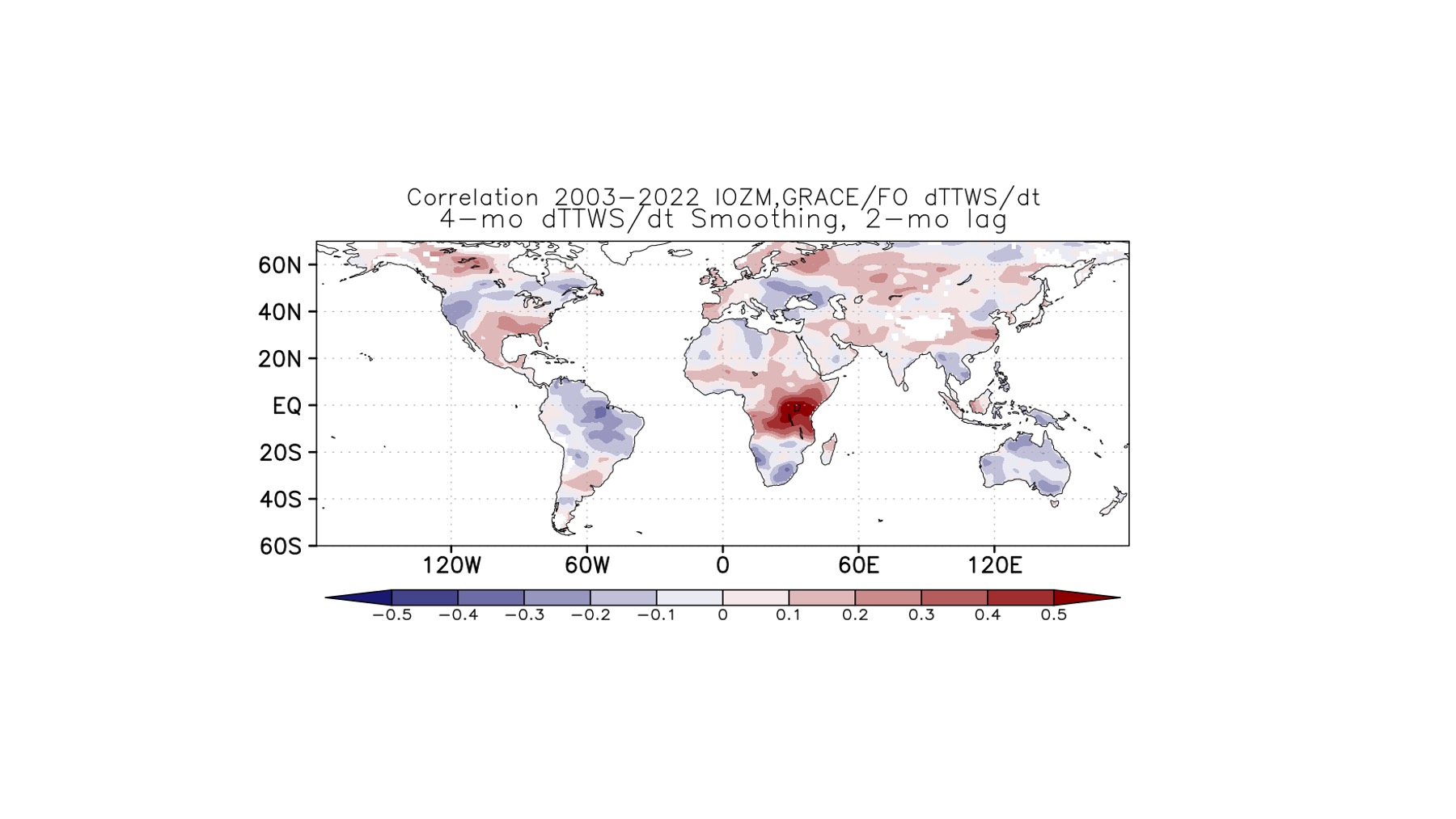


Figure S6. Correlation between the Indian Ocean Dipole (IOD) and the GRACE TWS tendency (d(TWS)/dt). This tendency was computed using centered differences, smoothed over four months and lagged 2 months with respect to the IOD. The latter was defined as area-average SST anomaly differences between regions: (50°E/70°E, 10S-10N) minus (90°E-110°E, 10°S-0°N).


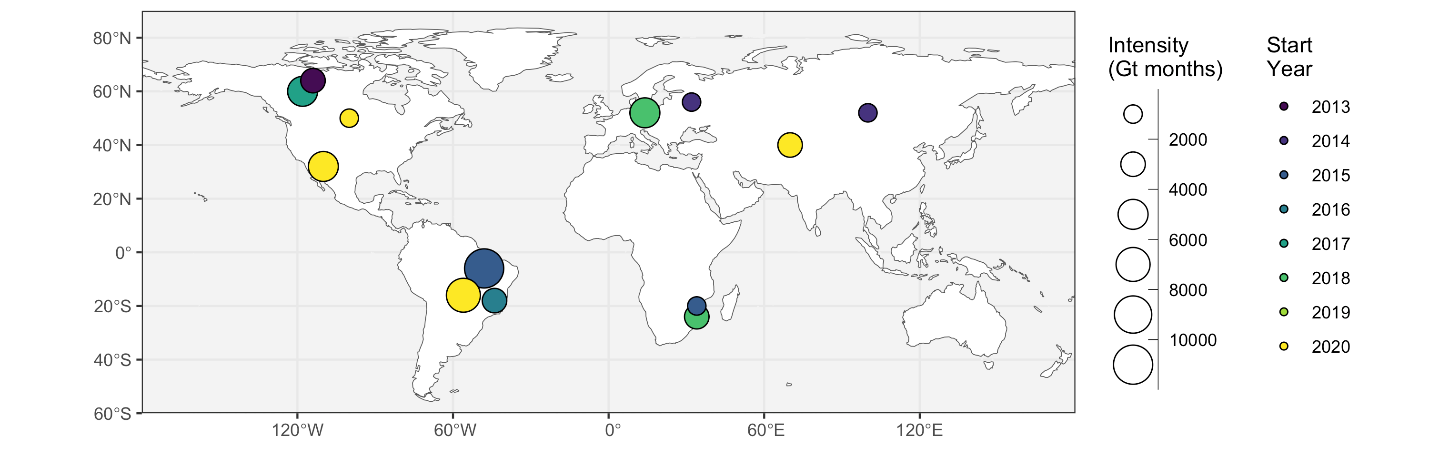


Figure S7. Intensity (circle size), starting year (color), and location (pinned to where the drought persisted longest) of major droughts that occurred concurrently or after the onset of the 2014-2016 abrupt decline in global TWS. Here we define major droughts as those within the top 30, in terms of intensity, of the 551 extreme dry events identified in the GRACE/FO record (2002-2021) by (Rodell and Li 2023).
